# Genome-Wide Association Study Based on Multiple Imputation with Low-Depth Sequencing Data: Application to Biofuel Traits in Reed Canarygrass

**DOI:** 10.1534/g3.115.017533

**Published:** 2015-03-12

**Authors:** Guillaume P. Ramstein, Alexander E. Lipka, Fei Lu, Denise E. Costich, Jerome H. Cherney, Edward S. Buckler, Michael D. Casler

**Affiliations:** * Department of Agronomy, University of Wisconsin-Madison, Madison, Wisconsin 53706; † Institute for Genomic Diversity, Cornell University, Ithaca, New York 14853; § Soil and Crops Section, School of Integrative Plant Science, Cornell University, Ithaca, New York 14853; †† Department of Plant Breeding and Genetics, Cornell University, Ithaca, New York 14853; ‡ International Maize and Wheat Improvement Center (CIMMYT), 56237 Texcoco, Mexico; ** Agricultural Research Service, United States Department of Agriculture, Ithaca, New York 14853; ‡‡ Agricultural Research Service, United States Department of Agriculture, Madison, Wisconsin 53706

**Keywords:** genome-wide association study, multiple imputation, genotyping by sequencing, bioenergy, *Phalaris* spp.

## Abstract

Genotyping by sequencing allows for large-scale genetic analyses in plant species with no reference genome, but sets the challenge of sound inference in presence of uncertain genotypes. We report an imputation-based genome-wide association study (GWAS) in reed canarygrass (*Phalaris arundinacea* L., *Phalaris caesia* Nees), a cool-season grass species with potential as a biofuel crop. Our study involved two linkage populations and an association panel of 590 reed canarygrass genotypes. Plants were assayed for up to 5228 single nucleotide polymorphism markers and 35 traits. The genotypic markers were derived from low-depth sequencing with 78% missing data on average. To soundly infer marker-trait associations, multiple imputation (MI) was used: several imputes of the marker data were generated to reflect imputation uncertainty and association tests were performed on marker effects across imputes. A total of nine significant markers were identified, three of which showed significant homology with the *Brachypodium dystachion* genome. Because no physical map of the reed canarygrass genome was available, imputation was conducted using classification trees. In general, MI showed good consistency with the complete-case analysis and adequate control over imputation uncertainty. A gain in significance of marker effects was achieved through MI, but only for rare cases when missing data were <45%. In addition to providing insight into the genetic basis of important traits in reed canarygrass, this study presents one of the first applications of MI to genome-wide analyses and provides useful guidelines for conducting GWAS based on genotyping-by-sequencing data.

Perennial crops, which include herbaceous energy crops (HEC), are increasingly studied as potential significant sources of energy because of their environmental benefits and the increase in prices of petroleum. In its 2005 billion-ton supply report, the US Department of Agriculture (USDA) and the US Department of Energy (USDOE) set the goal of a 30% replacement of US petroleum consumption with biofuels by 2030. This goal implies a production of approximately 1 billion dry matter tons of biomass per year from forest and agricultural lands. According to the report’s projections and assumptions, achieving that objective will require the following: (i) the conversion of active croplands, pasture land, and lands under the conservation reserve program (CRP) into perennial crop lands and (ii) achieving biomass yields ranging between 5.5 and 8 dry tons per acre from perennial crops (between 12.4 and 19.8 Mg ha^-1^) (US Department of Agriculture and US Department of Energy, 2005). Plant breeding has an important part to play in achieving such yields. To efficiently select for higher biomass yield, selection may act on secondary traits such as plant height and flowering time (Price and Casler 2014), resistance to biotic stress, tiller density (Boe and Beck 2008), leaf area, and plant architecture. Biomass quality considerations, for conversion into bioenergy, bring another suite of traits to bear in HEC breeding (*e.g.*, Vogel *et al.* 2011). Common methods for transforming biomass feedstock into energy include direct combustion, pyrolysis, and fermentation of sugars (soluble sugars, starch, cellulose, and hemicellulose) into ethanol (Wrobel *et al.* 2009).

Reed canarygrass (*Phalaris arundinacea* L., *Phalaris caesia* Nees) is a promising HEC in North America. It belongs to the tribe *Avenae* (sub-family *Pooideae*, family *Poaceae*), which includes the oat genus *Avena* (Quintanar *et al.* 2007). Of the grass model species, such as *Brachypodium distachyon* (Brachypodium), *Oryza sativa* (Rice), *Zea mays* (maize), and *Sorghum bicolor* (Sorghum), Brachypodium is the most closely related to reed canarygrass (Bouchenak-Khelladi *et al.* 2008). Reed canarygrass is a species complex that comprises two chromosomal races: the tetraploid race, *Phalaris arundinacea* L. (2n = 4x = 28), which is thought to be native to Europe, Asia, and North America (Jakubowski *et al.* 2012), and the hexaploid race, *Phalaris caesia* Nees (2n = 6x = 42) (McWilliam and Neal-Smith 1962; Baldini 1995). Most cultivars and wild accessions of *P. arundinacea* found in North America are of European ancestry (Casler *et al.* 2009a; Jakubowski *et al.* 2011). Reports of breeding efforts in *P. arundinacea* trace back to the early 20^th^ century in North America (Casler 2010), but this species was already cultivated in the 18^th^ century in Europe (Alway 1931). Some noncrop uses for reed canarygrass have been reported, such as phytoremediation (Picard *et al.* 2005), erosion control (Rice and Pinkerton 1993), and paper production (Pahkala and Pihala 2000). However, reed canarygrass has mostly been used as a forage crop. Consistently, most recent breeding efforts have focused on low alkaloid content for palatability to livestock, not on biomass yield (Wrobel *et al.* 2009).

Most of the research on HEC in the US has been focused on switchgrass (*Panicum virgatum* L.) (Sanderson *et al.* 1996), but reed canarygrass presents characteristics that may complement those of switchgrass; it is particularly tolerant to northern climates (Casler *et al.* 2009a), as well as to soil acidity, alkalinity, and moisture content (Bittman *et al.* 1980) and high levels of metals and minerals (Cureton *et al.* 1991). However, biomass yields in reed canarygrass are not very high. Based on trials in the US Midwest involving wild accessions and cultivars, genotype means for dry matter yield have been estimated to range from 7.6 to 10 Mg.ha^-1^ (Casler *et al.* 2009b). Also, quality tends to be lower in reed canarygrass than it is in switchgrass (Cherney *et al.* 1988). Nonetheless, judging from the significant genotypic variation observed in both biomass yield (Asay *et al.* 1968; Casler *et al.* 2009b) and quality (Carlson *et al.* 1996; Olmstead *et al.* 2013), improvement by selection of these primary biofuel traits is feasible.

Because no linkage map and no genome sequence are available in reed canarygrass, association studies have not been conducted in this species complex. However, genotyping by sequencing (GBS) provides the opportunity to call polymorphisms without prior marker development. With this technology, a genome-wide association study (GWAS) can be performed on reed canarygrass, but because no reference sequence is available, one limitation of such study is the inability to assign single nucleotide polymorphisms (SNP) to specific physical positions in the reed canarygrass genome. This has two important implications: (i) SNP-trait associations cannot be mapped to a particular region of the genome and (ii) no imputation method based on marker sequences, *e.g.*, hidden Markov models (HMM) or sliding-window algorithms, may be used. However, other methods are available for imputing unordered marker data (Poland *et al.* 2012; Rutkoski *et al.* 2013). Another limitation of using SNPs derived from GBS is the high amount of missing values in marker data and, therefore, the importance of accounting for uncertainty in the imputation of marker genotypes.

The purpose of this study was to make statistically sound inferences about associations between GBS markers with a high frequency of missing values and biofuel traits (related to biomass yield and quality) in reed canarygrass. GWAS was performed under the assumption of disomic inheritance in reed canarygrass, which is suggested by the alloploidy of a closely related species, *Phalaris aquatica* (Carlson *et al.* 1996). To perform statistically valid association tests, it was necessary to avoid false positives, not only by controlling for population structure and familial relatedness (Yu *et al.* 2006) but also by accounting for imputation uncertainty. Our study exemplifies the use of multiple imputation, initially proposed by Rubin (1987), to account for this source of variability when making inferences. Classification tree models, shown by Poland *et al.* (2012) and Rutkoski *et al.* (2013) to be promising, were used under this framework to impute missing values without information on markers’ genomic location and order. After a description of the variability present in the panels under study, we examine the general behavior of inferences in multiple imputation compared to more traditional methods that would not fully account for imputation uncertainty. Then, we present our results of GWAS performed in a multiple-imputation framework and describe the significant markers in light of where they map onto the *B. distachyon* genome.

## Materials and Methods

### Populations

Three panels were assessed in this study: two randomly segregating populations derived from biparental crosses (LP1 and LP2) comprising 177 and 189 clones, respectively (Supporting Information, Table S1), and one association panel (AP) comprising 590 clones originating from North America and Europe (Table S1). LP1 and LP2 were derived from two distinct crosses involving genotypes of WR00, a tetraploid cultivar originating from Wisconsin. In AP, four populations (AR Upland, Superior, PI-284179, and PI-236525) were accessions of *P. caesia* (hexaploid). The AP subset consisting of only the 550 *P. arundinaceae* clones is referred to as AP-4x.

Clones were assessed in spaced-plant trials arranged in a Sets-in-Reps design with two replicates. The trials were performed in two locations: Arlington, Wisconsin (43.3°N, 89.4°W) and Ithaca, New York (42.6°N, 76.4°W). Soil type was Plano silt loam (fine-silty, mixed, mesic Typic Argiudoll) in Arlington and was Niagara silt loam (fine-silty, mixed, active, mesic Aeric Endoaqualf) in Ithaca.

### Phenotypic data

Traits related to disease resistance (Ds), morphology [standability (St); leaf width (LW); leaf length (LL); total stem count (STC); plant height (PH); full height (FH)], phenology [heading date (HD); anthesis date (AD)], and quality [dry matter percentage (DM); neutral detergent fiber (NDF); acid detergent fiber (ADF); NDF digestibility (NDFD); acid insoluble ash (AIA); Klason lignin (Lignin); crude protein (CP); Ash (ASH); calcium (Ca); chlorine (Cl); copper (Cu); iron (Fe); potassium (K); magnesium (Mg); manganese (Mn); sodium (Na); phosphorus (P); sulfur (S); zinc (Zn); glucose (GLC); galactose (GAL); xylose (XYL); arabinose (ARA); GLC conversion efficiency (GLC_Eff); XYL conversion efficiency (XYL_Eff); energy content (BTU)] were determined on plants grown in Ithaca in 2009–2011 and in Arlington in 2010–2011 (Table 1). Quality traits were predicted from near-infrared reflectance spectroscopy (NIRS) measurement, using methodology described by Vogel *et al.* (2011). The NIRS prediction equations were developed on a diverse set of 110 reed canarygrass samples from the experiments described by Casler *et al.* (2009b). Wet-laboratory traits were determined for these samples using the procedures described by Vogel *et al.* (2011). The validity of predictions from the predictive models was verified by the extremely low frequency of outliers: nine biomass-quality samples with Mahalanobis distance more than three out of a total of >3300 samples (Shenk and Westerhaus 1991). The raw phenotypic data are available for download from http://dfrc.wisc.edu/sniper/.

**Table 1:** Summary of field traits and quality traits

Trait	Code	Unit	Environments	Comment
Disease	Ds	1–9	I09	Resistance to biotic stress
Standability	St	1–9	I09, I10	Resistance to lodging
Leaf width*^a^*	LW	Cm	I09, I11, A11	Component of leaf area
Leaf length*^a^*	LL	Cm	I09, I11	Component of leaf area
Total stem count	TSC	Count	I10, I11	Biomass yield component
Plant height*^a^*	PH	M	I09, I11, A11	Biomass yield predictive trait
Full height	FH	M	I09, I11, A11	Biomass predictive trait
Heading date	HD	DOY	I10, I11, A11	Length of vegetative stage
Anthesis date	AD	DOY	I10, I11, A11	Length of vegetative stage
Dry matter percentage*^b^*	DM	%	I11	Positively correlated with maturation and, therefore, with cellulose and lignin content (Dien *et al.* 2006)
Neutral detergent fiber*^b^*	NDF	%DM	I11	Lignin + celluloses + hemicelluloses
Acid detergent fiber*^b^*	ADF	%DM	I11	Lignin + cellulose
NDF digestibility*^b^*	NDFD	%	I11	Digestible fraction of NDF *in vitro* (Tilley and Terry 1963)
Acid insoluble ash*^b^*	AIA	%DM	I11	Positively correlated to DM digestibility (Van Keulen and Young 1977)
Klason Lignin*^b^*	Lignin	%DM	I11	Inhibits cellulosic fermentation (Vogel and Jung 2001)
				Increases conversion efficiency in thermochemical processes (Boateng *et al.* 2006)
Crude protein*^b^*	CP	%DM	I11	Protein + nonprotein nitrogen (excluding nitrate)
Ash content*^b^*	ASH	%DM	I11	Fouling of bioreactors and disposal costs (Brummer *et al.* 2002)
Calcium*^b^*	Ca	%DM	I11	
Chlorine*^b^*	Cl	%DM	I11	
Copper*^b^*	Cu	μg.g−1	I11	
Iron*^b^*	Fe	μg.g−1	I11	
Potassium*^b^*	K	%DM	I11	
Magnesium*^b^*	Mg	%DM	I11	
Manganese*^b^*	Mn	μg.g−1	I11	
Sodium*^b^*	Na	%DM	I11	
Phosphorus*^b^*	P	%DM	I11	
Sulfur*^b^*	S	%DM	I11	
Zinc*^b^*	Zn	μg.g−1	I11	
Glucose*^b^*	GLC	mg.g−1	I11	
Galactose*^b^*	GAL	mg.g−1	I11	
Xylose*^b^*	XYL	mg.g−1	I11	
Arabinose^b^	ARA	mg.g−1	I11	
GLC conversion efficiency*^b^*	GLC_Eff	%	I11	Expected fraction, on a mass basis, transformed into ethanol
XYL conversion efficiency*^b^*	XYL_Eff	%	I11	
Energy content*^b^*	BTU	BTU/kg	I11	1 Btu ≈ 1055 J

DOY, day of the year; %DM, percentage of dry matter; BTU, British thermal unit; A, Arlington (Wisconsin, USA); I, Ithaca (New York, USA). The two digits refer to the year.

a The trait was also measured in A10 and I10, in the LP1 and PL2 panels only.

b The trait was also measured in A10, in the LP1 and PL2 panels only.

Association analyses were performed on best linear unbiased estimations (BLUEs) of genotypes’ performance for a given trait, inferred from the following linear mixed model:

$$\begin{array}{l} y_{ijlmrc}=mean+genotype_{i}+location_{j}+year{\left(location\right)}_{jl}+rep.{\left(location\right)}_{jm}+{\left(\mathrm{year}\times \mathrm{rep}.\left(\mathrm{location}\right)\right)}_{jlm}+\\ {\left(genotype\times location\right)}_{ij}+{\left(genotype\times year\left(location\right)\right)}_{ijl}+plot{\left(year\left(location\right)\right)}_{jlrc}+\varepsilon_{ijlm} \end{array}$$

where $y_{ijlmrc}$ is the measurements at one of the 35 traits considered, $genotype_{i}$ is the genotypic value of clone *i*, modeled as fixed (to guarantee convergence of the fitting algorithm and to avoid assumptions about genotypes’ sampling). For all other terms, the corresponding effects were considered random, independent, and identically normally distributed: $location_{j}$ is the effect of location *j*; $year{\left(location\right)}_{jl}$ is the effect of year *l* within location *j*; $rep.{\left(location\right)}_{jm}$ is the effect of replicate *m* within location *j*; × indicates interactions; and $plot{\left(year\left(location\right)\right)}_{jlrc}$ is the effect of the plot at row *r* and column *c*, within an environment (year *l* within location *j*). Plot effects within environments were modeled as$\text{Normal}\left(\text{0},\left(\sum_{r}⊗\sum_{c}\right)\sigma_{\mathrm{plot}}^{2}\right)$, where $\sum_{r}⊗\sum_{c}$ is the Kronecker product of the first-order autoregressive covariance matrices on rows and on columns, respectively. The mixed models were fitted using ASREML-R (Butler *et al.* 2007).

The matrix of genotypes’ BLUEs, computed as described above, can be downloaded from http://dfrc.wisc.edu/sniper/.

### Marker data and quality control

Genome reduction, by *ApeKI* restriction, and sequencing were performed according to Elshire *et al.* (2011). Reduced DNA samples were sequenced on the Illumina HiSequation 2000, with 95 samples plus one negative control per lane. To simultaneously discover SNP markers and call genotypes, the UNEAK pipeline was used (http://www.maizegenetics.net/gbs-bioinformatics; Lu *et al.* 2013). This pipeline trims reads to a 64-bp length to limit sequencing errors and speedup computation, and discards markers based on a network filter designed to detect and eliminate markers showing complex relationships with others, which suggests paralogy and/or sequencing errors (Lu *et al.* 2013). A total of 29,313 SNPs were called out of the UNEAK pipeline. Marker genotypes were coded as allelic dosages: −1 for homozygotes at the reference allele, 0 for heterozygotes, and 1 for homozygotes at the alternate allele, assuming disomic inheritance in reed canarygrass (Carlson *et al.* 1996). The raw genotypic data are available for download from http://dfrc.wisc.edu/sniper/.

**Figure 1:**
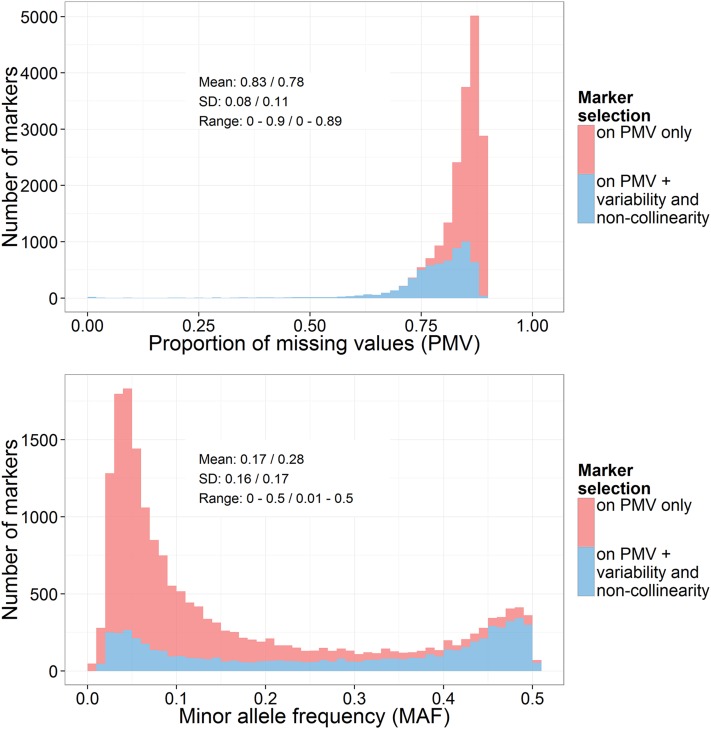
Summary statistics and distributions of proportions of missing values (PMV) and minor allele frequency (MAF) on markers retained after the first filtering step (“on PMV only”, *i.e.* PMV < 0.9 by panel; 18,818 markers) or after both filtering steps [“on PMV + variability and noncollinearity”, *i.e.* PMV < 0.9 by panel + filtering step recommended by van Buuren and Groothuis-Oudshoorn (2011), in which collinear and constant marker variables are discarded; 6138 markers]. Statistics are the mean, SD, and range for “on PMV only” / “on PMV + variability and noncollinearity”. Filtering for variability and noncollinearity preferentially discarded markers with high PMV and low MAF.

Markers were selected based on proportion of missing values (PMV) <0.90 in each of three panels, separately. It is typical to filter out GBS SNPs by a predetermined low missing rate (*e.g.*, PMV <0.20). However, we did not filter these markers to avoid removing potentially useful information, and also because a rigorous study of the behavior of the MI approach at different missing rates is merited. After this first filtering step, 18,818 markers were retained. Marker variables were then discarded if they met one of the following criteria: (i) being “constant”, *i.e.*, having a variance close to 0 (only 24 markers were discarded based on that criterion) or (ii) being “collinear”, *i.e.*, being correlated by at least 0.999 (in absolute value) to some other marker variable with a smaller amount of missing values in the dataset. This filtering step was recommended by van Buuren and Groothuis-Oudshoorn (2011) and was implemented in the mice R package. This avoided overly conservative tests in GWAS due to inadequate adjustment for multiple testing on highly correlated test statistics and also ensured that multiple imputations were not biased, as a result of strong correlations among predictors, and appropriately reflected imputation uncertainty. At this point, 6138 markers were considered for further analyses. Figure 1 shows the distributions of PMV and MAF for marker data after the first filtering step (18,818 markers) and after the second filtering step (6138 markers). As expected, filtering for variability and noncollinearity preferentially discarded markers with high PMV and low MAF. Note that filtering out markers on PMV <0.80 across panels (which is still a very lenient criterion) would have resulted in only 3419 markers being considered for subsequent analyses (Table S2). Also, for markers not meeting the criteria of van Buuren and Groothuis-Oudshoorn (2011), estimates of marker effects from averaged imputed data ((AD); see *Association analyses*) tended to show larger deviations from those obtained based on nonmissing data only ((CC); see *Association analyses*), no matter what the imputation uncertainty was (Table S3, Figure S2). Finally, effects of discarded markers as estimated from multiple imputes ((MI); see *Association analyses*) tended to show excessively strong shrinkage in comparison with (AD) estimates, with little adjustment of estimates in response to imputation uncertainty (Table S4, Figure S3).

The high values of PMV among selected markers (78% of missing values on average) reflect low sequencing depth of the GBS. From the quality control step, the marker data matrix X was produced, in which missing values were coded as NA. Matrix **X**, used as input to the imputation procedure, is available for download from http://dfrc.wisc.edu/sniper/.

### Marker data imputation

#### General principle of multiple multivariate imputation:

Consider some incomplete data set X, with $X_{\mathrm{obs}}$ and $X_{\mathrm{mis}}$ denoting the observed and missing data, respectively. Given an estimand of interest *θ* (*e.g.*, a model parameter), multiple imputation (MI) aims at producing a correct Monte Carlo (MC) approximation of $p\left(\theta |X_{\mathrm{obs}}\right)=\int p\left(\theta |X_{\mathrm{obs}},{\hat{X}}_{\mathrm{mis}}\right)p\left({\hat{X}}_{\mathrm{mis}}|X_{\mathrm{obs}}\right)d{\hat{X}}_{\mathrm{mis}}$ from *m* imputes of X, with ${\hat{X}}_{\mathrm{mis}}$ denoting some imputation of $X_{\mathrm{mis}}$. One implication of the MC approximation being correct is that, given an impute $\left\{X_{\mathrm{obs}},{\hat{X}}_{\mathrm{mis}}\right\}$, the distribution $p\left(\theta |X_{\mathrm{obs}},{\hat{X}}_{\mathrm{mis}}\right)$ is well-approximated, *i.e.*, the sampling model is correct. Another implication that is of particular concern when performing MI is that the distribution $p\left({\hat{X}}_{\mathrm{mis}}|X_{\mathrm{obs}}\right)$ is also well-approximated, *i.e.*, the imputation procedure is proper (Rubin 1987), which is defined as follows: let $\dot{\theta }$ be an estimate of *θ* from a given impute, let *W* be the within-impute estimation variance of $\dot{\theta }$, and let $B^{*}$ be the among-impute variance of $\dot{\theta }$ (corrected for a finite number of imputes): (i) $\text{E}\left[\bar{\theta }|X\right]=\hat{\theta }$, *i.e.*, the average of $\dot{\theta }$ over imputes $\left(\bar{\theta }\right)$ is an unbiased estimator of $\hat{\theta }$, the estimate of *θ* from the hypothetically complete dataset; (ii) $\text{E}\left[\bar{W}|X\right]=W$; and (iii) $\text{E}\left[B^{*}|X\right]\geq \text{Var}\left(\bar{\theta }\right)$ (Rubin 1987; van Buuren 2012). Condition (i) implies correct assumptions regarding the imputation model and the source of missingness in the data. Typically, the missing-at-random assumption (no factor other than those accounted for in the imputation model caused missingness) (Rubin and Little 2002) is necessary to guarantee that condition (i) holds. Conditions (ii) and (iii) imply that inferences from MI are confidence-valid (van Buuren 2012), *i.e.*, that the total variance of $\dot{\theta }$ is realized in a conservative way: $\text{E}\left[\hat{\text{Var}\left(\dot{\theta }\right)}\right]\geq W+\text{Var}\left(\bar{\theta }\right)$. In this study, we assess imputation models for unbiasedness, under specific assumptions [condition (i)], but we did not test imputation models for their ability to preserve variability in the data [condition (ii)] or to correctly reflect among-impute variability [condition (iii)].

When performing MI on data sets containing missing values at several variables (SNP markers), one must sample from the joint distribution of missing values at all variables. To achieve this, two strategies have been proposed: joint modeling (JM) and fully conditional specification (FCS). JM consists of sampling from the joint distribution directly, by ordinary MC (Rubin and Schafer 1990; Schafer 2010). This method is theoretically sound, but it requires complex model specifications and assumptions that, if not correct, may result in imputation bias. JM cannot accommodate tree-based approaches that have the advantage of being flexible and not requiring any model specification. FCS, however, implicitly samples from some hypothetical joint distribution by repeatedly sampling from the fully conditional distributions at each variable of interest using a Gibbs sampling scheme (van Buuren *et al.* 2006; van Buuren 2007). Because it relies on Markov chain MC (MCMC), FCS can be computationally costly. Also, there is no guarantee that the joint distribution actually exists, so FCS is usually described as a pseudo-Gibbs sampling procedure (Gelman and Raghunathan 2001; van Buuren *et al.* 2006; van Buuren 2007). However, simulation studies have suggested that FCS is robust to such issues (van Buuren *et al.* 2006). A critical incentive for using FCS rather than JM is that FCS allows for much more flexibility in the imputation procedure: each fully conditional distribution can be derived separately, using either parametric (*e.g.*, linear regression) or nonparametric models (*e.g.*, classification trees). This flexibility is particularly valuable when dealing with missing values at many variables, in which case JM may simply not be practical (Gelman and Raghunathan 2001).

#### Implementation of multiple imputation:

In this study, we sampled missing values from $p\left({\hat{X}}_{\mathrm{mis}}|X_{\mathrm{obs}}\right)$ by FCS. In the pseudo-Gibbs sampling process, missing values at the $k^{\text{th}}$ SNP were sampled from $p\left({\hat{X}}_{\mathrm{mi}s_{k}}|X_{\mathrm{obs}},{\hat{X}}_{\mathrm{mi}s_{-k}}\right)$ based on a classification and regression tree model (CART) (Breiman *et al.* 1984). For unbiasedness, imputation of $X_{\mathrm{mis}}$ relied on the missing-completely-at-random (MCAR) assumption, stating that missingness occurred at random, with no factor causing SNP genotypes to be systematically missing (Rubin and Little 2002). CART presents the advantages of not requiring explicit model specification and conveniently accommodating nonlinear effects.

The general algorithm was:

For impute r=1,…,m:

1. For k=1,…,q: fill in missing values at the $k^{\text{th}}$ marker by random draws ${\hat{X}}_{}^{\left(r,0\right)}{}_{\mathrm{mi}s_{k}}$ from $X_{{\mathrm{obs}}_{k}}$.
2. For l=1,…,L:
  For k=1,…,q:
  sample ${\hat{X}}_{}^{\left(r,l\right)}{}_{\mathrm{mi}s_{k}}$ from
    $$p\left({\hat{X}}_{\mathrm{mi}s_{k}}|X_{\mathrm{obs}},{\hat{X}}_{\mathrm{mi}s_{1}}^{\left(r,l\right)}\mathrm{,\ldots ,}{\hat{X}}_{\mathrm{mi}s_{k-1}}^{\left(r,l\right)},{\hat{X}}_{\mathrm{mi}s_{k+1}}^{\left(r,l-1\right)}\mathrm{,\ldots ,}{\hat{X}}_{\mathrm{mi}s_{q}}^{\left(r,l-1\right)}\right)$$
3. Return $\left\{X_{\mathrm{obs}},{\hat{X}}_{\mathrm{mis}}^{\left(r,L\right)}\right\}$ as ${\dot{X}}^{\left(r\right)}$,

where *m* is the total number of imputes of X, *q* is the number of marker variables, and *L* is the number of MCMC iterations.

The number of imputes *m* was set to 20, based on available computational and memory resources. The number of iterations *L* up to which the actual sampling occurred (*i.e.*, the burn-in period) was set to 10, based on previous studies (Dai *et al.* 2006; Burgette and Reiter 2010) and available computational resources.

MI, based on CART, was implemented according to Doove *et al.* (2014) with packages mice (van Buuren and Groothuis-Oudshoorn 2011) and rpart (Therneau and Atkinson 1997) in the R programming language (R Development Core Team 2014). CARTs were fitted with pruning up to at least five donors per terminal node (*i.e.*, no less than five observations at each leaf in the tree). For the MI algorithm to be computationally tractable, only a subset of SNPs was considered for fitting the CART models: for each SNP *k*, only the 500 SNPs showing the highest marginal mutual information with SNP *k* were considered as potential predictors.

MI implemented as described above took 1 d per imputation (chain of 10 iterations) on a workstation consisting of 24 Intel Xeon X7460 CPU processors at 2.66 GHz with 264 Gb of RAM. The procedure was parallelized on five (m/4) threads.

Two types of marker data were generated from the multiple imputation procedure: the MI set of matrices $\dot{X}=\left\{{\dot{X}}^{\left(1\right)}\mathrm{,\ldots ,}{\dot{X}}^{\left(m\right)}\right\}$ and the average-dosage (AD) matrix $\bar{X}=\mathrm{av}g_{r}\left({\dot{X}}^{\left(r\right)}\right)$ (*i.e.*, each element in $\bar{X}$ was the mean of genotype codes across the m=20 imputes). The array $\dot{X}$ and the matrix $\bar{X}$ from the imputation procedure can be downloaded from http://dfrc.wisc.edu/sniper/.

### Association analyses

#### Data subsets:

Only tetraploid samples were considered for GWAS, and the following subsets of the data were examined separately when testing marker-trait associations: LP1, LP2, and AP-4x (AP panel with only *P. arundinacea* samples) consisting of n=177,189,550 individuals, respectively. After discarding marker variables with MAF <0.05 (as estimated from $X_{\mathrm{obs}}$), in each subset separately, 5024, 5096, and 5228 markers were assayed in association testing, respectively. Figure S1 shows, for each subset separately, the distribution of PMV and MAF for markers selected prior to conducting GWAS. The distributions are equivalent across subsets and similar to that observed with the larger set of 6138 markers, with the exception that the average MAF increased from 0.28 to 0.32.

#### Association model:

Within each of the three data subsets (LP1, LP2, and AP-4x), the linear mixed model of Yu *et al.* (2006) was fitted for each SNP *k* retained, using the P3D (population parameters previously determined) approximation for computational efficiency (Zhang *et al.* 2010; Kang *et al.* 2010):

$$g_{\mathrm{obs}}=\mu +X_{{\mathrm{obs}}_{k}}\beta +{\bar{Q}}_{\mathrm{obs}}v+Z_{\mathrm{obs}}u+e_{\mathrm{obs}}\left(\text{CC}\right)$$

$$g=\mu +{\bar{X}}_{k}\beta +\bar{Q}v+\mathrm{Zu}+e\left(\text{AD}\right)$$

$$g=\mu +{\dot{X}}_{k}\beta +\bar{Q}v+\mathrm{Zu}+e\left(\text{MI}\right)$$

$$u∼\mathrm{Normal}\left(\mathrm{0,}\bar{K}\sigma_{u}^{2}\right);e\mathrm{∼Normal}\left(\mathrm{0,}I\sigma_{e}^{2}\right)$$

$$g=\mu +{\dot{X}}_{k}\beta +\dot{Q}v+\mathrm{Zu}+e\left({\text{MI}}^{*}\right)$$

$$u\mathrm{∼Normal}\left(\mathrm{0,}\dot{K}\sigma_{u}^{2}\right);e\mathrm{∼Normal}\left(\mathrm{0,}I\sigma_{e}^{2}\right)$$

where $g=\left\{\mathrm{genotyp}e_{i}\right\}$ is the *n*-vector of clones’ genotypic values as described above; $X_{k}$, ${\bar{X}}_{k}$, and ${\dot{X}}_{k}$ are the vectors of allelic dosage for SNP *k* and correspond to the three types of marker data described above; β is the effect of SNP *k*; **Q** is the matrix of the first *t* components used to account for population structure (Price *et al.* 2006) obtained from a principal component analysis (PCA) performed either on $\bar{X}\left(\bar{Q}\right)$ or $\dot{X}\left(\dot{Q}\right)$; **v** is the vector of their effects; **Z** is the design matrix for relating observations to clones; **u** is the vector of random polygenic effects; **K** is the realized genetic relationship matrix estimated from either $\bar{X}\left(\bar{K}\right)$ or $\dot{X}\left(\dot{K}\right)$; **e** is the vector of residuals; and **I** is the identity matrix. $\sigma_{u}^{2}$ and $\sigma_{e}^{2}$ were estimated by restricted maximum likelihood (REML). The R package rrBLUP (Endelman 2011) was used to calculate the realized relationship matrix **K** and estimate $\sigma_{u}^{2}$ and $\sigma_{e}^{2}$. Subscript obs refers to the subset of individuals for which there was no missing value at SNP *k*.

(CC) is the analysis restricted to complete cases (nonmissing values) for a given marker tested, with the approximation of **Q** and **K** estimated from $\bar{X}$ used as fixed, to account for population structure and relatedness. In (AD), marker effects are assessed from $\bar{X}$, considered as fixed. In (MI) and (MI*), marker effects are assessed from $\dot{X}={\dot{X}}^{\left(1\right)}\mathrm{,\ldots ,}{\dot{X}}^{\left(m\right)}$ and the variability across imputes $\dot{X}$ is accounted for. Relying on the MCAR assumption, (CC) served as a reference for assessing the consistency of estimates from (AD) or (MI): (AD) estimates departing too much from (CC) estimates were considered unreliable, especially when imputation uncertainty (*γ*; see below) was high. Estimates from (MI) were expected to show appropriate control over imputation uncertainty, *i.e.*, shrinkage toward zero as *γ* increases. In (MI*), marker effects are assessed from $\dot{X}$ as in (MI), but variability across imputes for estimates of **Q** and **K** is also accounted for, through $\dot{Q}$ and $\dot{K}$, thus allowing for full control over imputation uncertainty in inferences regarding *β*. Although (CC), (AD), and (MI) are convenient to assess the impact of imputation uncertainty on *β* estimates, (MI*) should be the method permitting the most statistically sound inferences regarding marker-trait associations.

In AP-4x, population structure and relatedness were, respectively, accounted for by one principal component and by matrix $\bar{K}$ calculated on AP-4x individuals. In LP1 and LP2, no principal component and no genetic relationship matrix were included in the GWAS model, then equivalent to a single-marker analysis model.

#### Combination of parameter estimates in MI and calculation of p values:

In (CC) and (AD), significance of marker-trait associations was assessed by performing an F-test for the regression coefficient estimate *β*, as described in Kang *et al.* (2008). In (MI) and (MI*), an F-test was performed in which the *p* value was (Rubin and Schenker 1986; van Buuren 2012):

$$\mathrm{Pr}\left(F_{1,v}>\frac{{\bar{\beta }}^{2}}{T}\right)$$

where $\bar{\beta }=\frac{1}{m}\sum_{r=1}^{m}{\hat{\beta }}^{\left(r\right)}$ is the average estimate of*β* over imputations, with ${\hat{\beta }}^{\left(r\right)}$ being the estimate of *β* based on the $r^{\text{th}}$ imputation; $T=\bar{W}+B^{*}$ is the (estimated) total variance of *β* estimates, partitioned into $\bar{W}=\frac{1}{m}\sum_{r=1}^{m}\text{Var}\left({\hat{\beta }}^{\left(r\right)}\right)\dot{=}E\left[\text{Var}\left(\hat{\beta }|{\dot{X}}^{\left(r\right)}\right)\right]$, the average within-impute variance of *β* estimates, and $B^{*}=\frac{m\mathrm{+1}}{m}{\text{Var}}_{r}\left({\hat{\beta }}^{\left(r\right)}\right)\dot{=}\text{Var}\left(\text{E}\left[\hat{\beta }|{\dot{X}}^{\left(r\right)}\right]\right)$, the among-impute variance of *β* estimates, corrected by $\frac{m+1}{m}$ for unbiasedness ($\dot{=}$means “asymptotically equal”). Barnard and Rubin (1999) derived a formula for the number of denominator degrees of freedom *v* of the F-statistic:

$$v=\frac{v_{m}v_{obs}}{v_{m}+v_{obs}}$$

where $v_{m}=\frac{m-1}{\lambda^{2}}$, with $\lambda =\frac{B^{*}}{T}$; $v_{obs}=\frac{v_{com}+1}{v_{com}+3}v_{com}\left(1-\lambda \right)$, with $v_{com}=n-\left(2+t\right)$ being the number of degrees of freedom for the hypothetically complete dataset.

For a particular coefficient *β*, the uncertainty in its estimate due to imputation is characterized by *γ*, the fraction of information about *β* lacking due to missingness:

$$\gamma =\frac{s+\frac{2}{v+3}}{1+s}$$

with $s=\frac{B^{*}}{\bar{W}}$ (Rubin 1987; van Buuren 2012). *γ* reflects the dependency of the inferences about *β* on the imputation procedure. Although quite complex, the *γ*-statistic may simply be interpreted as *λ*, the proportion of among-impute variance in inferences, adjusted for a finite number of imputes (van Buuren 2012). In this study, $\gamma =1.026\lambda \left(R^{2}=1\right)$. According to Li *et al.* (1991), 0≤γ<0.2, 0.2≤γ<0.3, and 0.3≤γ<0.5 indicate a “modest,” “moderately large,” and “high” missing data problem, respectively.

#### False discovery rate and significance for marker-trait association:

The *p* values obtained from (CC), (AD), or (MI) were transformed into adjusted false discovery rates (FDR) using the method of Storey and Tibshirani (2003). Marker-trait associations for which FDR <0.1 in (CC) or (MI*) were deemed significant and considered for further analyses, although evidence from (MI*) was preferred because (CC) relied on the approximation of **Q** and **K** estimated from $\bar{X}$ used as fixed in the GWAS model and also because (CC) was considered more prone to false positives or false negatives due to smaller sample sizes, $n_{obs}<n$ (see *Discussion*). The R package qvalue (Dabney *et al.* 2013) was used to compute FDR.

## Results

### Genotypic variability in the panels

#### Phenotypic traits:

Traits were measured in varying numbers of years and locations (Table 1). Some traits were measured in only one location (Ds, TSC, St, and Quality traits in AP, measured/predicted in Ithaca only) and/or during only 1 yr (Ds only in 2009 and Quality traits only in 2011). As a result, they would show an inflated genotypic variance. Ds, St, LL, LW, and all quality traits showed a much higher ratio of genotypic-to-phenotypic variance (H) in AP compared to LP1 and LP2. PH showed a higher H in LP1 and AP compared to LP2. AD showed a higher H in LP2 and AP compared to LP1 (Table 2).

**Table 2:** Summary statistics on the field traits (9) and quality traits (26) in each panel

Trait	Mean			Total SD			H		
	LP1	LP2	AP	LP1	LP2	AP	LP1	LP2	AP
Ds	7.0	6.3	4.8	2.0	2.6	2.7	0.49	0.71	0.71
TSC	97.5	122.6	69.1	61.5	54.2	75.8	0.33	0.33	0.30
St	6.7	6.1	6.5	1.6	1.8	1.9	0.19*^a^*	0.10*^a^*	0.66
LW	21.0	18.6	17.0	6.0	3.9	3.8	0.16	0.16	0.29
PH	109	109	99	35.8	32.4	42.9	0.31	0.06	0.22*^a^*
LL	224	229	243	67.3	67.4	85.3	0.11	0.14	0.30
FH	162	160	143	31.2	28.5	36.3	0.31	0.24	0.32
HD	153	153	153	7.2	6.5	6.5	0.30	0.33	0.38
AD	157	158	158	7.4	6.5	7.0	0.27	0.45	0.43
DM	95.9	95.8	95.8	0.37	0.36	0.32	0.10	0.12	0.49*^a^*
CP	13.6	13.4	8.7	7.3	7.3	2.0	0.09	0.03	0.49
ADF	38.9	38.5	40.5	5.2	5.3	2.9	0.08	0.03	0.71
NDF	64.8	64.6	65.8	5.6	5.5	3.9	0.02	0.09	0.79
Lignin	5.4	5.6	6.5	1.83	1.84	0.58	0.12	0.05	0.55
NDFD	57.5	57.3	48.3	14.4	13.6	5.3	0.18	0.09	0.59
ASH	8.0	7.9	6.8	2.6	3.4	0.9	0.09	0.06	0.37
AIA	3.7	3.9	4.0	1.14	1.07	0.88	0.12	0.08	0.43
Ca	0.33	0.33	0.26	0.14	0.14	0.04	0.14	0.00	0.30*^a^*
P	0.24	0.25	0.2	0.151	0.07	0.031	0.11	0.01	0.55
Mg	0.24	0.25	0.18	0.162	0.151	0.058	0.04	0.05	0.48
K	1.9	1.9	1.6	0.93	0.35	0.35	0.26	0.18	0.69
Na	0.013	0.012	0.012	0.001	0.002	0.001	0.00*^a^*	0.00	0.45
S	0.25	0.24	0.20	0.20	0.07	0.05	0.20	0.02	0.48
Cl	0.69	0.67	0.62	0.32	0.26	0.18	0.00	0.03	0.38
Fe	122	163	156	69.9	69.5	92.1	0.17*^a^*	0.00	0.51
Mn	70.7	68.9	68.1	52.2	11.8	15.9	0.14	0.00	0.46
Zn	27.1	28.3	27.0	3.2	3.1	2.4	0.00*^a^*	0.00	0.52
Cu	4.9	4.3	3.7	4.2	3.7	1.9	0.21	0.19	0.51
BTU	7790	7823	7748	163.1	170.1	93.8	0.10	0.10	0.39
GLU	309	312	324	77.4	36.7	17.5	0.06	0.03	0.58*^a^*
XYL	216	216	216	6.4	7.2	7.7	0.00	0.05	0.72
ARA	34.7	33.4	33.3	6.6	6.3	3.6	0.22	0.05	0.54
GAL	26.2	26.1	23.6	6.4	4.9	1.6	0.16	0.03	0.43
GLC Eff.	55.6	56.4	48.4	11.4	12.6	5.9	0.05	0.01	0.41*^a^*
XYL Eff.	59.6	59.6	68.2	9.4	8.9	9.7	0.17	0.21	0.70

H, broad-sense heritability (in LP1 and LP2) or proportion of genotypic variance to phenotypic variance (in AP), calculated on an individual-plant basis, as an estimate of $\frac{\sigma_{G}^{2}}{\sigma_{G}^{2}+\sigma_{GE}^{2}+\sigma_{e}^{2}}$, with $\sigma_{G}^{2}$ the genotypic variance, $\sigma_{GE}^{2}$ the variance of genotype-by-environment interactions, and $\sigma_{e}^{2}$ the residual variance, from the model described in *Phenotypic data* in *Material and Methods*, but fitted within each panel separately and with genotype as a random effect.

a The spatial correlation model did not converge for the corresponding trait and panel. If the model did not converge, then a simpler model, not including the plot(year(location)) effect, was fitted.

#### Population structure:

**Figure 2:**
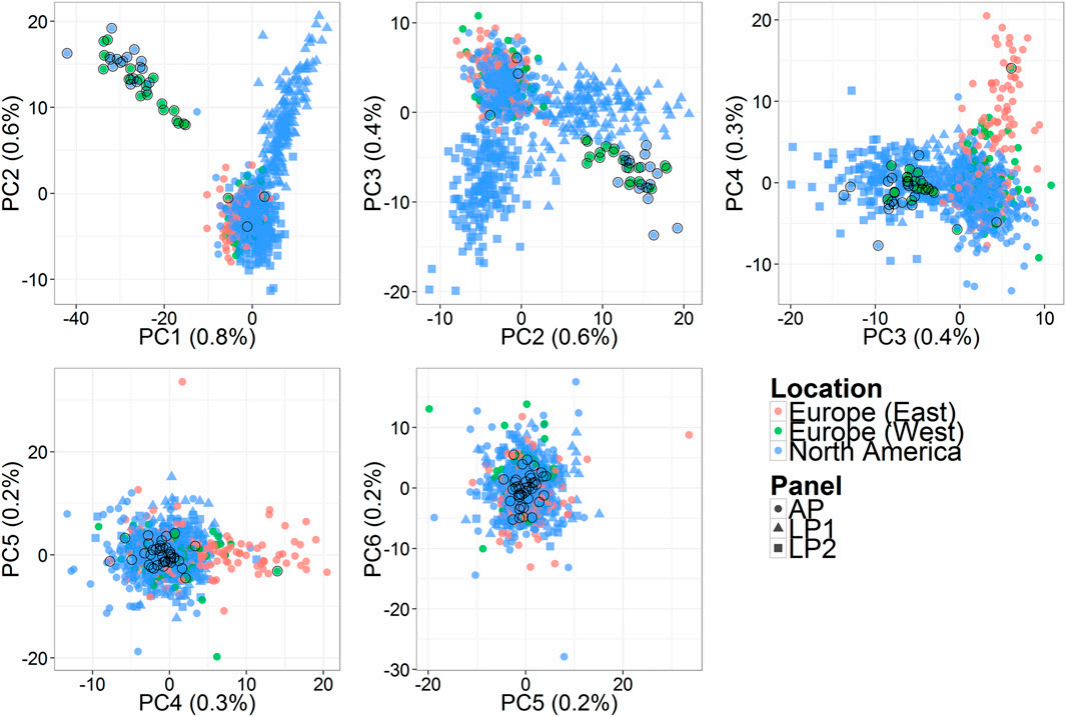
Principal component analysis (PCA) on the three panels combined: first six components and the proportion of marker variation explained, in parentheses on axis labels. Colors refer to location of origin (as in Table S1). Shapes refer to the panel (as in Table S1). Data points for *P. caesia* clones are circled in black.

To analyze population structure, principal component analysis (PCA) was performed on the average dosage matrix $\bar{X}$. For the whole dataset (panels LP1, LP2, and AP combined), the first four principal components (PC) were deemed relevant for describing population structure based on proportions of variance explained and grouping patterns on PCs (Figure 2). Grouping patterns in the whole dataset are consistent with race (first PC, distinguishing accessions that are *Phalaris arundinacea* from those that are *Phalaris caesia*), panel (second and third PCs), and geographical origin—Eastern Europe *vs.* North America or Western Europe (fourth PC). The fifth and sixth PCs do not seem to reflect any grouping and explain a small proportion of the total genotypic variation compared with the first four PCs.

PCA was also performed for four other subsets of the data: LP1, LP2, AP, and AP-4x (AP panel with only *P. arundinacea* samples). The numbers of selected PCs for those subsets were 0, 0, 2, and 1, respectively (data not shown). The relevant PCs in AP were consistent with race and geographical origin, respectively, in the same way as in the whole data set. The relevant PC in AP-4x was consistent with geographical origin.

### Analysis of imputation uncertainty

#### Consistency of PCs and genetic relationship coefficients across imputes:

Consistency of a variable in MI is defined as the correlation with the same variable in (AD), in absolute value, averaged over imputes. Such value aims at assessing to what extent it is appropriate to use **Q** and **K**, estimated from $\bar{X}$, as fixed, in (CC), (AD), and (MI) association analyses. In the whole dataset, **K** estimates seem very consistent over imputes (consistency of 0.90 ± 0.0002; Table 3), but although estimates of $Q_{1}$ (first PC) are quite consistent, the estimates at the subsequent PCs lose coherence across imputes, with the fourth PC having a consistency of only 0.043 (± 0.032). This result suggests that accounting for imputation uncertainty with respect to population structure variables—when those are estimated from marker data—is important, which implies that (MI*) should be the most appropriate type of analysis for assessing marker-trait associations. In the AP-4x subset, the consistency of the only relevant PC is 0.076 (± 0.067) and the consistency of genetic relationship coefficients is 0.934 (± 0.0002).

**Table 3:** Consistency of population structure and genetic relationship variables across imputes

Variable	Average Correlation with (AD) estimate	SD Across Imputes
${\dot{Q}}_{1}$	0.74	0.026
${\dot{Q}}_{2}$	0.48	0.088
${\dot{Q}}_{3}$	0.21	0.100
${\dot{Q}}_{4}$	0.04	0.032
${\dot{Q}}_{5}$	0.05	0.039
${\dot{Q}}_{6}$	0.05	0.034
$\dot{K}$	0.90	0.0002

Consistency of confounder variables across imputes is reflected here by $avg_{r}\left\{\left|Cor\left({\dot{Q}}_{ij}^{\left(r\right)},{\bar{Q}}_{ij}\right)\right|\right\}$ for principal component j (j=1,…,6) and $avg_{r}\left\{\left|Cor\left({\dot{K}}_{ij^{\prime }}^{\left(r\right)},{\bar{K}}_{ij^{\prime }}\right)\right|\right\}$ for realized genetic relationship (with $\left(i,i^{\prime }\right)$ a given pair of genotypes). Cor is the Pearson correlation.

#### Consistency across imputation schemes of marker effects and significance:

#####  From complete case (CC) to average dosage (AD):

**Figure 3:**
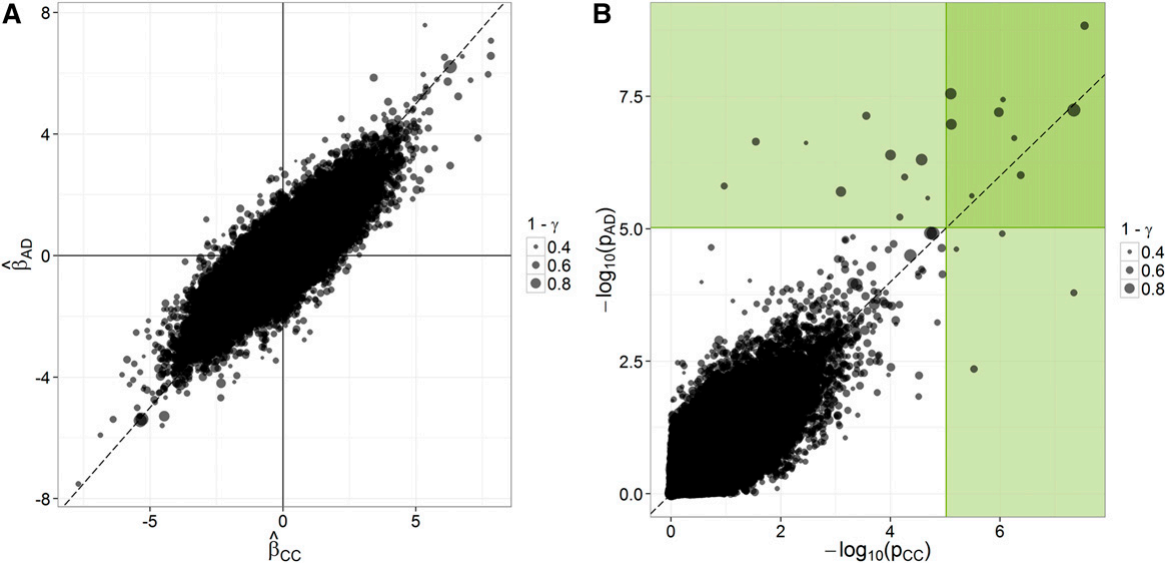
Concordance in inferences from (CC) to (AD) in the AP-4x subset of the data. The points’ size refers to the stability of inferences across imputes (1−γ). (A) Concordance across procedures and over all traits in marker effect estimates, standardized within each trait. (B) Concordance across procedures and over all traits in significance [−log_10_(*p*)]; green areas correspond to significant associations according to a Bonferroni correction on a single-trait basis (*p* < 9.56 × 10^−6^).

Under the MCAR assumption, the relationship between $X_{\mathrm{obs}}$ and the trait of interest adequately reflects the effect of markers in the whole sample. That is, for any marker-trait association, the estimate ${\hat{\beta }}_{CC}$ based on complete cases is unbiased with respect to $\hat{\beta }$, the estimate of *β* based on the hypothetically complete data set. It follows from the MCAR assumption that imputations are unbiased if estimates of marker effects from (AD), ${\hat{\beta }}_{AD}$, are unbiased with respect to those from (CC), ${\hat{\beta }}_{CC}$. The plot (Figure 3A) and the regression analysis (Table 4) of ${\hat{\beta }}_{AD}$ on ${\hat{\beta }}_{CC}$, across traits and markers, strongly suggest that there is no bias from (CC) to (AD). For marker-trait associations with low imputation uncertainty (γ<0.2), there is a close relationship between ${\hat{\beta }}_{CC}$ and ${\hat{\beta }}_{AD}$ (R^2^ = 0.94). However, for marker-trait associations with moderate-to-high imputation uncertainty (γ>0.2), large differences between ${\hat{\beta }}_{CC}$ and ${\hat{\beta }}_{AD}$ can be observed, but at random. Such differences indicate noise in inferred values of *β* and opportunities for false positives or false negatives (Figure 3B).

**Table 4:** Regression analysis of the relationship between ${\hat{\beta }}_{CC}$ and ${\hat{\beta }}_{AD}$

Selection on ***γ***	Intercept		Slope		${\hat{\sigma }}_{e}^{2}$ (R^2^)
	Coefficient Estimate (± SE)	*p*	Coefficient Estimate (± SE)	*p*	
None	0.0010 (±0.0011)	0.34	1.00 (± 0.0012)	<0.0001	0.238 (0.76)
γ<0.2	−0.0024 (±0.0048)	0.61	0.99 (± 0.0053)	<0.0001	0.048 (0.94)
γ>0.2	0.0011 (±0.0011)	0.33	1.00 (± 0.0012)	<0.0001	0.240 (0.76)

Model: ${\hat{\beta }}_{CC}=\text{Intercept}+\text{Slope}.{\hat{\beta }}_{AD}+e.$ The estimated regression coefficients suggest no systematic bias from ${\hat{\beta }}_{CC}$ to ${\hat{\beta }}_{AD}$ (Slope = 1). However, for associations with values of *γ* above 0.2, inferences tend to be more erratic, as indicated by the substantially higher residual variance $\left({\hat{\sigma }}_{e}^{2}\right)$ at γ=0.2. The model meets the assumptions of linearity but not normality of residuals. Although *p* values are not exact, they are provided for information. *β* estimates are from analyses on the AP-4x subset.

##### From average dosage (AD) to multiple imputation (MI):

**Figure 4:**
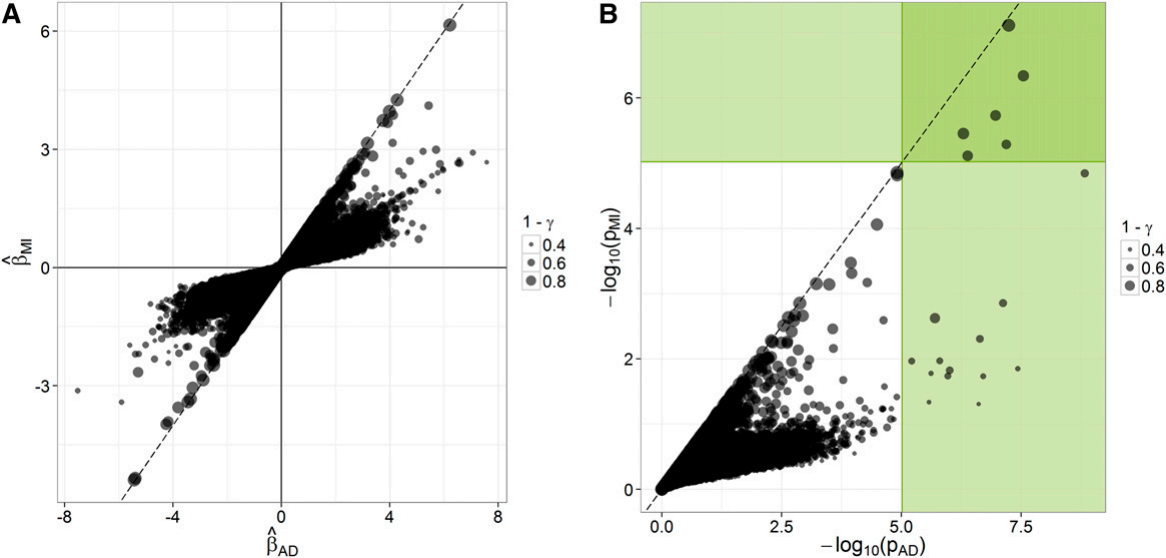
Concordance in inferences from (AD) to (MI) in the AP-4x subset of the data. The points’ size refers to the stability of inferences across imputes (1−γ). (A) Concordance across procedures and over all traits in marker-effect estimates, standardized within each trait. (B) Concordance across procedures and over all traits in significance [−log_10_(*p*)]; green areas correspond to significant associations according to a Bonferroni correction on a single-trait basis (*p* < 9.56 × 10^−6^).

The purpose of MI is to make sound inferences when one bases statistical analyses on imputed data. Therefore, MI should produce more conservative estimates ${\hat{\beta }}_{MI}\left(\bar{\beta }\right)$ when imputation uncertainty regarding marker-trait associations is high, in order to avoid declaring as significant associations that are due to “fortuitous” imputations. The plot (Figure 4A) and the regression analysis (Table 5) of ${\hat{\beta }}_{MI}$ on ${\hat{\beta }}_{AD}$, across traits and markers, show that as *γ* increases, estimates of *β* tend to shrink toward zero, from (AD) to (MI). This behavior results in higher *p* values (lower significance) for those associations in which imputation uncertainty is high: whether associations inferred in (AD) are true or false, their *p* values will tend to fall above the significance threshold in (MI) if their among-impute variability is too large, but there is good agreement between (AD) and (MI) for low values of *γ* (Figure 4B). Only for γ=0 (no variation among impute) would there be no adjustment on ${\hat{\beta }}_{MI}$ and $\text{Var}\left({\hat{\beta }}_{MI}\right)$. In other words, one should rely on (AD) for inferences only if it can be assumed that the situation is “close enough” to the case where γ=0. Clearly, this is not the case for these data. In Figure S4, the consistency of marker effects from (CC) to (MI) is shown.

**Table 5:** Regression analysis of the relationship between ${\hat{\beta }}_{AD}$ and ${\hat{\beta }}_{MI}$

Effect	Coefficient Estimate (±SE)	*p*	R^2^
Intercept	−0.0048 (±0.0028)	0.0943	0.91
$b_{1}:{\hat{\beta }}_{MI}$	1.0073 (±0.0069)	<0.0001	
$b_{2}:\gamma$	0.0097 (±0.0061)	0.1152	
$b_{3}:{\hat{\beta }}_{MI}\times \gamma$	5.7864 (±0.0162)	<0.0001	

Model: ${\hat{\beta }}_{AD}=\text{Intercept}+\left(b_{1}+b_{3}\gamma \right).{\hat{\beta }}_{\mathrm{ΜΙ}}+b_{2}.\gamma +e.$ The estimated regression coefficients suggest no difference between ${\hat{\beta }}_{AD}$ and ${\hat{\beta }}_{MI}$ for associations for which $\gamma =0\left(b_{1}=1\right)$, and shrinkage of ${\hat{\beta }}_{MI}$ toward 0 as *γ* increases $\left(b_{3}>0\right)$. The model meets the assumptions of linearity but not normality of residuals. Alhough *p* values are not exact, they are provided for information. *β* estimates are from analyses on the AP-4x subset.

##### From multiple imputation (MI) to multiple imputation with full account of imputation uncertainty (MI^*^):

**Figure 5:**
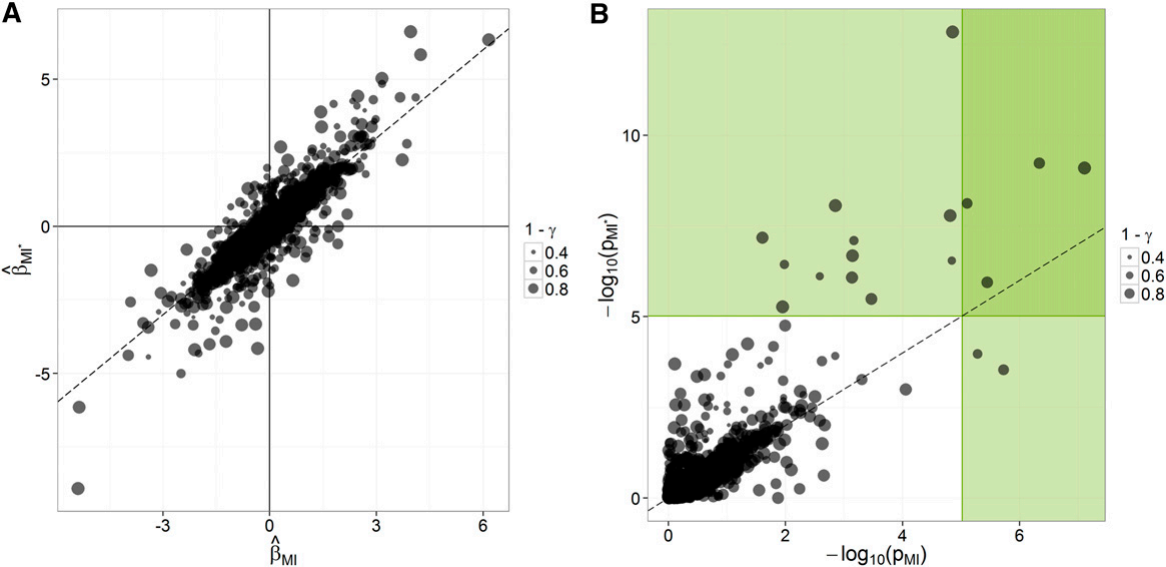
Concordance in inferences from (MI) to (MI*) in the AP-4x subset of the data. The points’ size refers to the stability of inferences across imputes (1−γ). (A) Concordance across procedures and over all traits in marker-effect estimates, standardized within each trait. (B) Concordance across procedures and over all traits in significance [−log_10_(*p*)]; green areas correspond to significant associations according to a Bonferroni correction on a single-trait basis (*p* < 9.56 × 10^−6^).

**Figure 6:**
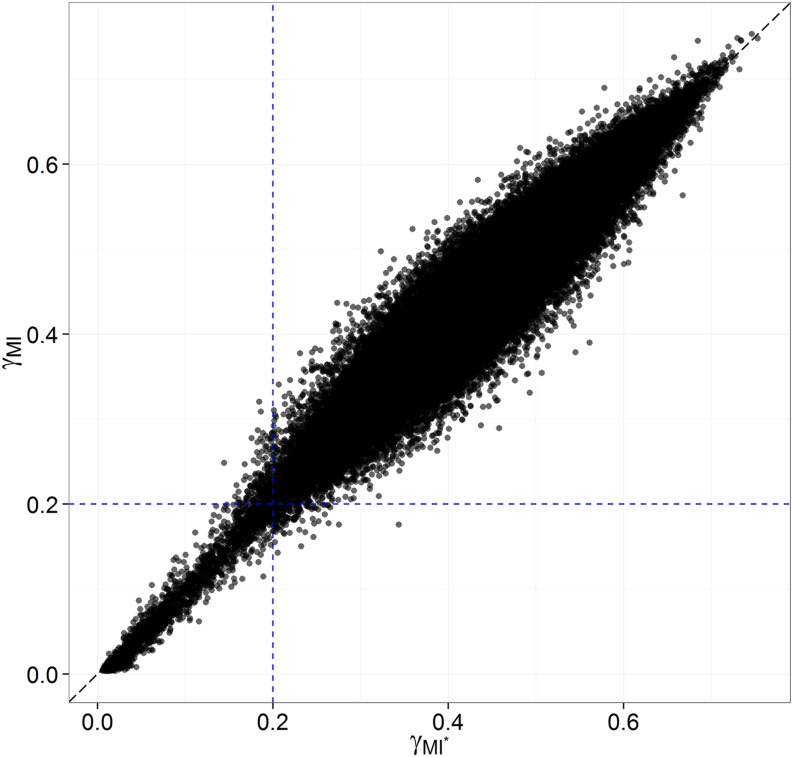
Concordance in imputation uncertainty, as reflected by the γ-statistic, over all traits in the AP-4x subset from (MI*) to (MI). The blue dashed lines correspond to the seemingly critical threshold of γ > 0.2, beyond which the γ-statistic loses coherence across procedures.

In (MI*), the marker data matrix **X**, but also the principal component and the genetic relationship matrices **Q** and **K** (both estimated from **X**) are allowed to vary over imputes. Although considering **Q** and **K** as fixed (estimated by $\bar{Q}$ and $\bar{K}$**)** was convenient for studying the behavior of inferences from (CC) to (AD) to (MI), (MI*) should permit the safest inferences by appraising the imputation uncertainty in the imputed genotypes as well as in the resulting **Q** and **K** estimates. Figure 5A suggests concordance of *β* estimates from (MI) to (MI*), despite the relatively low consistency of **Q** estimates across imputes (Table 3). Also, marker effects with low imputation uncertainty seem less shrunk toward zero in (MI*), where the variability in **Q** and **K** estimates is accounted for. As a result, more significant associations could be detected in (MI*) than in (MI) (Figure 5B). Values of *γ* were quite consistent from (MI) to (MI*), with increased noise around perfect concordance for values of *γ* above 0.2 (Figure 6).

#### Imputation uncertainty and potential gains in power:

**Figure 7:**
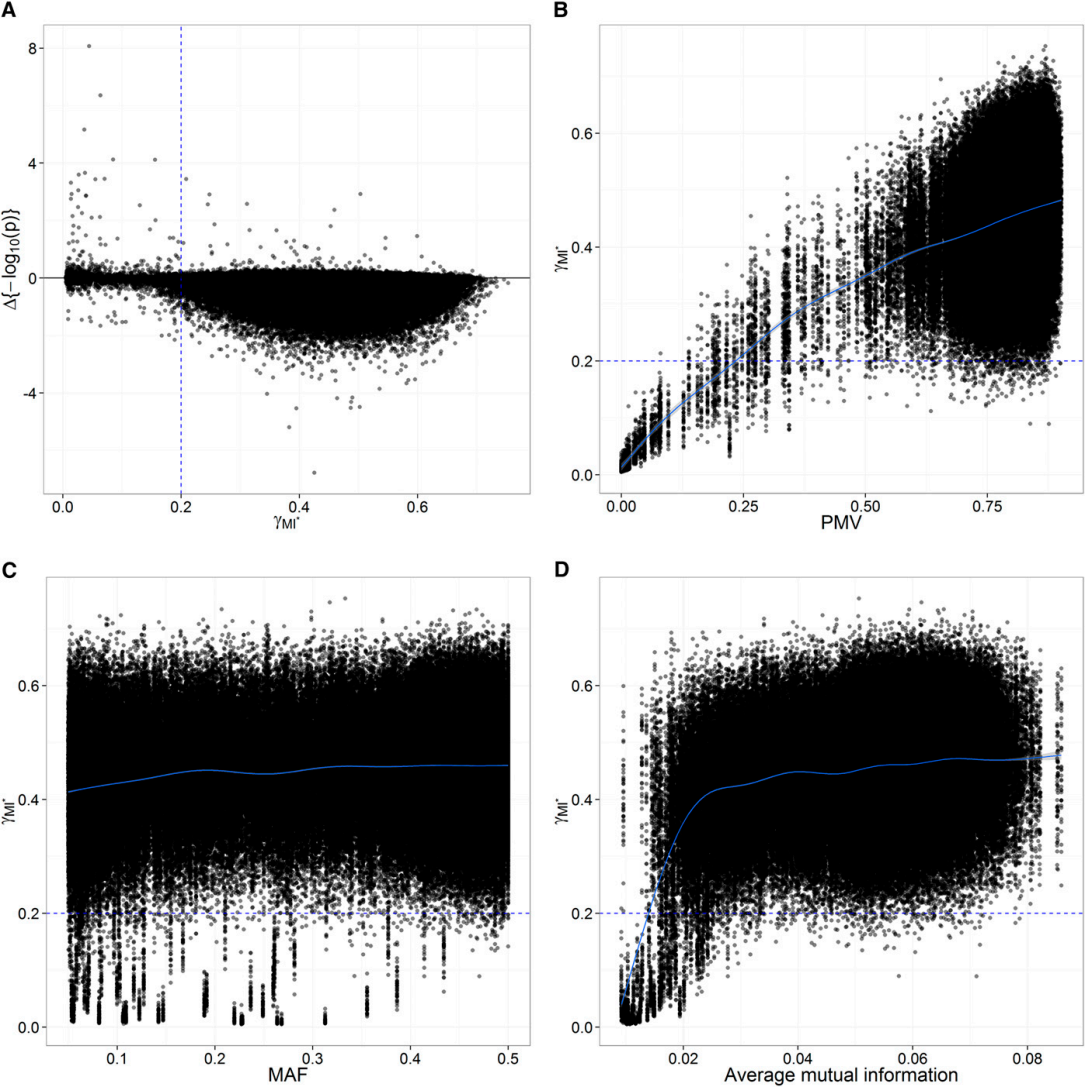
(A) Relationship between γ, in (MI*), and the difference in significance from (CC) to (MI*) [Δ{−log_10_(*p*)}]. Decrease in significance [−log_10_(*p*)] seems to occur more often and with higher intensity for γ > 0.2; presumably, there would to be more opportunities for gaining detection power with γ < 0.2. (B–D) Potential factors affecting imputation uncertainty (γ): relationship between γ, in (MI*), and (B) PMV (proportion of missing values), (C) MAF (minor allele frequency), and (D) the average mutual information between one given marker and all other markers in the dataset. In purple are the smoothed curves obtained from thin plate regression (default smoother in mgcv R package; Wood 2003). The γ-statistics and *p* values are from analyses on the AP-4x subset.

As Figure 7A suggests, for γ<0.2 (*i.e.*, markers with a modest missing data problem) there were generally small differences in significance $\left(-\log_{10}\left(p\right)\right)$ from (CC) to (MI*), but some opportunity for a few outstanding gains in significance, with increases of up to 8.08 in $-\log_{10}\left(p\right)$ (for the association between marker TP87762 and TSC; see next paragraph and Table 7). Assuming that the associations detected are true positives, this increase in significance may be considered an increase in power. This higher sensitivity in GWAS would logically come from a higher effective sample size due to imputation, with a modest missing data problem at the same time. For γ>0.2, however, there was little benefit from imputation, with very few opportunities for higher sensitivity from (CC) to (MI*): most values for the differential in $-\log_{10}\left(p\right)$ are actually negative. Those results show again that a threshold of 0.2 for *γ* is a good guideline for setting apart inferences with excessively high imputation uncertainty.

#### Potential factors influencing imputation uncertainty:

Because there is good consistency between *γ* and the apparent usefulness of imputation for increasing power, it would be helpful to identify the factors that allow the analyst to effectively predict *γ* and determine how likely imputation is to generate gains in power. Figure 7, B–D, respectively, show the marginal relationships between *γ* and proportion of missing values (PMV), MAF, or the average mutual information (AMI) between given markers and all other markers in the data set. There seems to be a strong positive relationship between *γ* and PMV, with values of *γ* that are below 0.2 for markers that have up to approximately 25% of missing values. As PMV increases, the slope for *γ* decreases and the variability around the conditional mean, as determined by a smoothing curve, increases. There seems to be a weak correlation between *γ* and MAF, with markers having high MAF showing slightly higher imputation uncertainty, especially for MAF <0.1. However, this relationship might be an artifact from markers with low PMV, which tended, by happenstance, to have lower MAF. There seems to be some (nonlinear) relationship between *γ* and AMI, but it is not clear whether it is due to the fact that markers with very low AMI tended to have lower PMV on average: for AMI <0.02, average PMV is 0.45 (SD 0.32); for AMI >0.02, average PMV is 0.80 (SD 0.071). A simple additive model fitted to the data with arcsin(γ) regressed on PMV, MAF, and AMI suggests that that all three factors considered have a significant effect on imputation uncertainty: MAF (usually equivalent to marker genotype variance) and—to a much larger extent—PMV generate imputation uncertainty, whereas AMI reduces imputation noise (Table 6). The model fitted has some predictive value (r^2^_CV_, prediction reliability in 10-fold cross-validation, was 0.21), but a rather large part of the variation could not be accounted for (Table 6).

**Table 6:** Regression analysis on potential factors affecting imputation uncertainty

Effect	Coefficient Estimate (±SE)	*p*	r^2^_CV_ (±SD)	MSE (±SD)
Intercept	0.0934 (± 0.0018)	< 2.2e-16	0.21 (± 0.0064)	0.00687 (± 8.7e-05) [Null model: 0.00873 (± 1.3e-04)]
$b_{1}:$ PMV	0.4806 (±0.0029)	< 2.2e-16		
$b_{2}:$ MAF	0.0099 (±0.0025)	8.3e-05		
$b_{3}:$ AMI	−0.1789 (± 0.0325)	3.6e-08		

Model: $\text{arcsin}\left(\gamma \right)=\text{Intercept}+b_{1}.PMV+b_{2}.MAF+b_{3}.AMI+e.PMV:$ Proportion (between 0 and 1) of missing values. The null model is arcsin(γ)=Intercept+e. The arcsin transformation was used to account for γ∈[0;1]. The model meets the assumptions of linearity and normality of residuals, but not the assumption of homoscedasticity: variance of residuals tends to increase as $\hat{\gamma }$ increases. The *γ*-statistics are from analyses of the AP-4x subset. MAF, minor allelle frequency (between 0 and 1); AMI, average mutual information (base 2); r^2^_CV_, squared coefficient of correlation in 10-fold cross-validation; MSE, mean squared error in 10-fold cross-validation.

### Association analyses

#### Significance of marker-trait associations:

Significant associations, for which FDR <0.1 in (CC) or (MI*), were detected in the AP-4x subset and in the LP2 subset; no significant association was detected in LP1. These involved nine markers (thereafter “significant markers”): TP140584; TP184396; TP191264; TP217634; TP268059; TP341988; TP477925; TP521945; and TP87762. The one association involving TP341988 was detected only in LP2 (TP341988 was not included in the analysis in AP-4x due to the threshold of MAF >0.05), whereas the others were detected only in AP-4x. As shown in Table 7, behavior of inferences from (CC) to (MI*) was highly dependent on *γ*: for values of *γ* above 0.3 (associations involving TP184396, TP191264, TP217634, TP268059, TP341988, and TP521945), marker effects estimated from (MI*) were closer to zero and significance of associations decreased, certainly as a result of the shrinkage of marker effects as well as the high among-impute variance. These marker-trait associations that lost significance from (CC) to (MI*) generally had a high PMV (above 0.69). However, associations with γ<0.3 (involving TP140584, TP477925, and TP87762), characterized by lower PMV (below 0.34), were more prone to show higher significance from (CC) to (MI*), with similar (sometimes larger) estimated marker effects. In some cases, such as TP140584-TSC, TP477925-ARA, TP477925-Mg, TP477925-Ds, TP477925-P, TP477925-TSC,TP87762-ARA, and TP87762-Mg, associations were deemed significant in (MI*) but not in (CC). Such behavior suggests an increase in detection power despite the uncertainty associated with imputation. Those associations generally show consistency from (CC) to (MI*), which would bring evidence for unbiasedness of the imputation procedure. However, for associations TP477925-Mn (γ=0.17) and TP477925-Cu (γ=0.09), marker effects were estimated to be weaker in (MI*). Under the assumption that the imputation procedure is proper, such results suggest that the associations detected in (CC) were actually false positives.

**Table 7:** Results of association analyses based on (CC) and (MI*) procedures

SNP	Trait	*p* (FDR)		$\hat{\beta }$		*γ*	MAF	PMV
		(CC)	(MI*)	(CC)	(MI*)			
TP140584	Ds	2.9e-08 (0.00011)	2.9e-07 (0.00075)	1.6	1.3	0.24	0.065	0.34
	Mg	1.2e-05 (0.060)	7.8e-07 (0.0014)	0.022	0.022	0.28		
	TSC	0.00029 (0.38)	3.6e-07 (0.00063)	−17	−22	0.25		
TP184396	Lignin	3.2e-05 (0.082)	0.15 (1)	−0.18	−0.046	0.58	0.41	0.73
TP191264	AD	4.5e-08 (0.00023)	0.27 (1)	2.1	0.19	0.43	0.12	0.83
	HD	3e-06 (0.016)	0.47 (1)	1.8	0.12	0.38		
	K	1.4e-05 (0.036)	0.2 (1)	0.21	0.031	0.49		
	CP	3e-05 (0.052)	0.33 (1)	0.95	0.096	0.35		
	Lignin	3e-05 (0.082)	0.47 (1)	−0.32	−0.027	0.46		
TP217634	K	3.3e-06 (0.017)	0.0033 (1)	−0.26	−0.17	0.61	0.08	0.74
TP268059	CP	2.5e-05 (0.052)	0.095 (1)	−0.74	−0.2	0.46	0.14	0.76
	XYL_Eff	3.1e-05 (0.081)	0.1 (1)	2.9	0.78	0.43		
TP341988	GLC*^a^*	1.6e-05 (0.084)	0.034 (1)	−13	−5.6	0.5	0.11	0.69
TP477925	Mn	1.1e-06 (0.0053)	0.00011 (0.56)	5.6	3.7	0.17	0.067	0.22
	Cu	7.8e-06 (0.041)	0.00029 (1)	−0.68	−0.44	0.09		
	St	7.9e-06 (0.040)	5.9e-10 (3.1e-06)	−0.64	−0.73	0.085		
	PH	2.7e-05 (0.069)	1.1e-06 (0.006)	−5.8	−5.3	0.063		
	ARA	9.9e-05 (0.25)	7.6e-09 (4e-05)	1	1.3	0.16		
	Mg	0.00022 (0.45)	8.1e-08 (0.00021)	0.015	0.02	0.21		
	Ds	0.00041 (0.26)	3.3e-06 (0.0057)	0.84	0.9	0.049		
	P	0.0023 (0.96)	8.4e-07 (0.0044)	0.0073	0.0096	0.055		
	TSC	0.15 (0.97)	6.7e-08 (0.00018)	−5.3	−17	0.063		
TP521945	CP	4.2e-07 (0.0022)	0.015 (1)	1.4	0.47	0.39	0.074	0.78
	GAL	5.5e-07 (0.0028)	0.017 (1)	1.1	0.41	0.5		
	XYL_Eff	8.9e-07 (0.0046)	0.018 (1)	−5.3	−2	0.49		
	P	9.2e-07 (0.0047)	0.03 (1)	0.021	0.0075	0.49		
	ARA	6.3e-06 (0.032)	0.045 (1)	2.2	0.81	0.54		
	AD	1.1e-05 (0.029)	0.024 (1)	1.9	0.73	0.41		
	PH	2.7e-05 (0.069)	0.048 (1)	−10	−3.7	0.46		
	K	5.5e-05 (0.096)	0.027 (1)	0.23	0.089	0.34		
TP87762	Ds	4.5e-08 (0.00011)	8e-10 (4.2e-06)	1.9	1.9	0.027	0.055	0.015
	TSC	1.7e-05 (0.088)	1.4e-13 (7.5e-10)	−23	−38	0.044		
	St	1.8e-05 (0.046)	1.6e-08 (4.3e-05)	−0.88	−1	0.026		
	ARA	0.00097 (0.50)	2.1e-07 (0.00056)	1.3	1.8	0.037		
	Mg	0.0013 (0.94)	8.7e-09 (4.6e-05)	0.019	0.031	0.036		

All results presented are based on the AP-4x subset of the data (AP panel with only *P. arundinacea* samples), except the association between TP341988 and GLC, for which the results presented are based on the LP2 panel (in the AP panel, TP341988 has MAF <0.05). $\hat{\beta }$, Estimated additive effect of the nonreference allele relatively to the reference allele; ***γ***, Imputation uncertainty; FDR, false discovery rate, as in Storey and Tibshirani (2003); MAF, minor allele frequency; PMV, proportion of missing values.

a One exception is the association between TP341988 and GLC, for which the results presented are based on the LP2 panel (in the AP panel, TP341988 has MAF <0.05).

**Figure 8:**
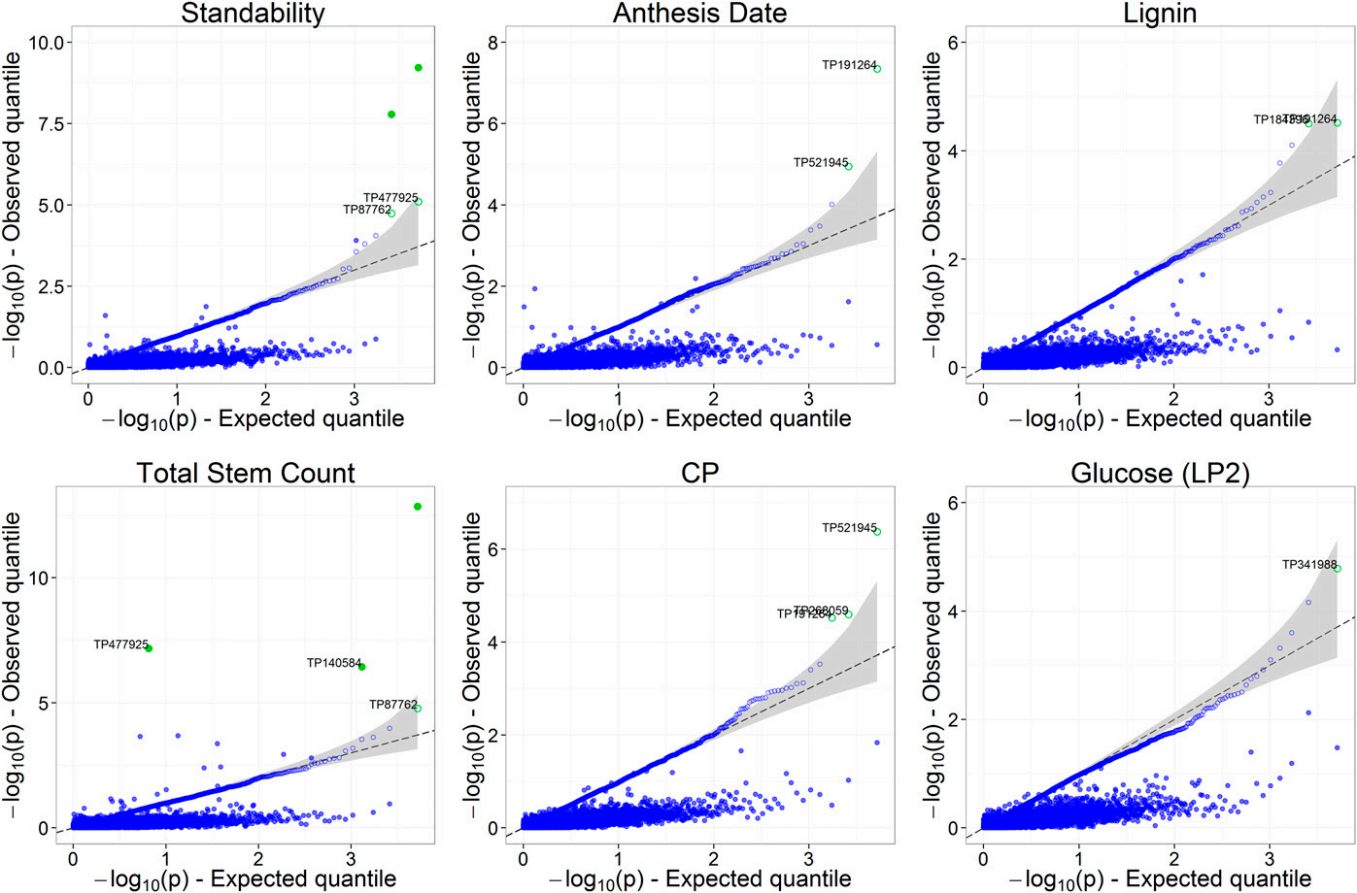
Q-Qplot of *p* values from (CC), for St, TSC, AD, CP, Lignin (in AP-4x subset), and GLC (in LP2 subset), with corresponding *p* values from (MI*). Expected quantiles are from the (CC) analysis; the corresponding values from (MI*) are shown unordered to reflect how consistent quantiles are across analyses. Open circles, −log_10_(*p*) based on (CC); full circles, −log_10_(*p*) based on (MI*). Green symbols indicate associations for which FDR <0.1. Gray areas correspond to the 95% C.I. of quantiles under the null hypothesis that *p* values follow a *Uniform*(0,1). The *p* values from (CC) seem to follow a *Uniform*(0,1), indicating good control for potential confounders (population structure and relatedness). However, *p* values from (MI*) do not follow a *Uniform*(0,1) because of losses of significance caused by imputation uncertainty.

The good overall concordance between observed and expected quantiles of *p* values for (CC) suggests that potential confounders (due to population structure and relatedness in AP-4x) were well accounted for in the GWAS models (Figure 8). Regarding (MI*), the strong overall deflation of $-\log_{10}\left(p\right)$ suggests that *p* values simply do not follow a Uniform(0,1) distribution under the null hypothesis (no significant marker-trait association) because of the extra variability due to imputation uncertainty, which was large for the majority of markers (Figure 6).

#### Correlation among significant markers:

**Figure 9:**
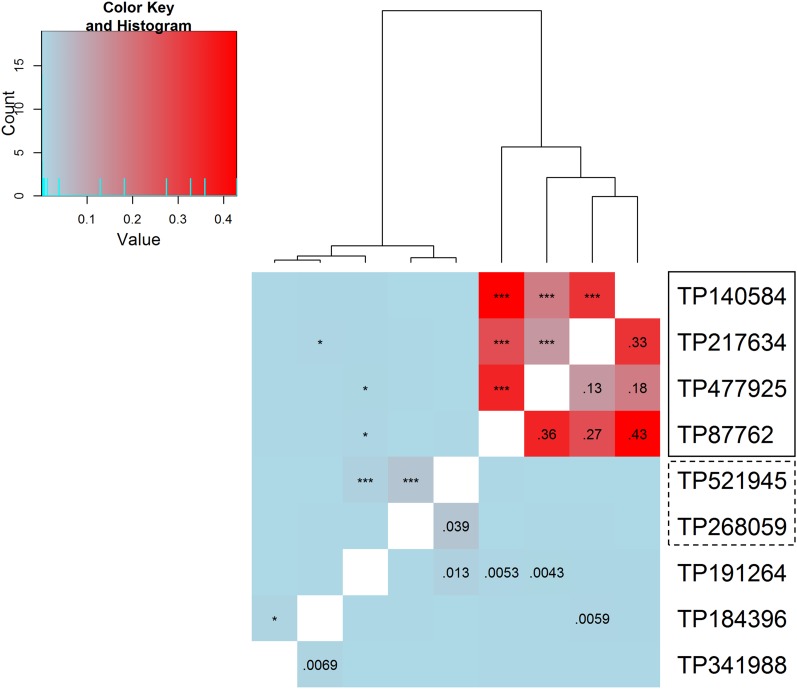
Squared coefficients of correlation among significant markers based on $\bar{X}$ (below diagonal elements). Above diagonal elements indicate significance; *: *p* < 0.05; **: *p* < 0.01; ***: *p* < 0.001. TP140584, TP217634, TP477925, and TP87762 form a group of fairly correlated marker loci. TP521945 and TP268059 are in very mild correlation with each other. TP191264, TP184396, and TP341988 each seem independent of other significant markers.

In the absence of haplotypic information, the squared correlation between markers’ allelic dosages $\left(r^{2}\right)$ was used to reflect linkage disequilibrium (LD) between markers. The $r^{2}$ values were calculated based on $\bar{X}$. Among the nine significant markers, there seemed to be one group of four markers in moderately strong association with each other (TP140584, TP217634, TP477925, and TP87762) and one group of two markers in mild association (TP521945 and TP268059) (Figure 9). This grouping is consistent with the associations inferred from GWAS (*i.e.*, markers in one group tend to be associated to similar traits; Table 7), except for TP217634, which is not associated with the same traits as TP140584, TP477925, or TP87762.

#### Homology of SNP 64-bp sequences with the Brachypodium distachyon genome:

For each marker, a 64-bp read containing the corresponding SNP was obtained from the UNEAK pipeline. The reads corresponding to the nine significant markers were analyzed for homology with the *Brachypodium distachyon* genome (v1.0) (Table 8). Three of the nine marker reads significantly match regions of the *Brachypodium distachyon* genome: (i) TP184396, shown to be negatively associated to Lignin (the nonreference allele has a negative effect on the trait), is in a region homologous to the last intron of an arginine-tRNA ligase gene (*e*-value = 1.2E-6); (ii) TP268059, shown to be negatively and positively associated with CP and XYL_Eff, respectively, is in a region homologous to the only exon in a phenylalanine/histidine-ammonia-lyase gene (*e*-value = 3.4E-12); and (iii) TP341988, shown to be negatively associated with GLC in LP2, is in a region homologous to the second exon of a translation elongation factor G gene (*e*-value = 4.3E-19). The moderate significance of TP184396's alignment to the *B. distachyon* genome may be due to the fact that the marker is located in an intron, which is usually more likely than exons to be under low selection pressure and diverge substantially across species.

**Table 8:** Sequence information on significant markers

SNP	Associated Trait(s)	Sequence Read	Location in *Brachypodium distachyon* (Transcript ID — Annotation: Putative Protein Function)	% Identity [% Coverage] (*e*-value)
TP140584	TSC, Ds, Mg	CAGCCCGGCAGTTTGGTCTTGGGCAAGTATCTCCCCATTTCTTCCTCCATCAC**C/T**TAACAGAGAG	—	—
TP184396	Lignin	CAGCCTTATTCACCCACAATTC**C/T**AAAAGTTGTGCATAAATTTGCACGCTCCTAGTGCTCAACTC	Chr. 2: 34773626-34777668 (BRADI2G34680.1 — Intron 17: Arginine-tRNA ligase)	91% [68%] (1.2E-6)
TP191264	AD, HD, K, CP, Lignin	CAGCGACAAAACTCTCAAGGA**C/T**CACTCGTGATTTAGGCAACCACCACAGCACTTAGCTGAAAAA	—	—
TP217634	K	CAGCGCGTTCTCCTTCCTTCCTGCAACCTCTAGTAGCCTCCCTGCAAATCAATCCGACGGA/TAAC	—	—
TP268059	CP, XYL_Eff	CAGCTCAGAGCAATACGAGGCCATGGCGATTTC**C/G**GCTCCCTTCAAGCCATAGTCCAAGCTCGGG	Chr. 3: 48837936-48837999 (BRADI3G47120.1.1 — Exon 1: Phenylalanine/Histidine-Ammonia-Lyase)	89% [100%] (3.4E-12)
TP341988	GLC	CTGCAATTTGGAA**A/T**GCAAGGACACTTGAATCAACATCATGGTAGGAGCCATCAACCAGCACTGA	Chr. 5: 20217343-20221405 (BRADI5G16980.1 — Exon 2: Translation Elongation Factor G)	94% [98%] (4.3E-19)
TP477925	St, ARA, TSC, Mg, P, Ds, PH	CTGCGGATTC**A/C**ACCCTTACTAGGCGATAGCTCTGATCTATACCTTTCCTAGGAGAGACCACTTC	—	—
TP521945	CP, GAL, XYL_Eff, P, ARA, AD, PH, K	CTGCTC**C/T**TGCCGGCGTGGTGCGTGGCTCCCGTTGCCGCTGAAAAAAAAAAAAAAAAAAAAAAAA	—	—
TP87762	TSC, Ds, St, Mg, ARA	CAGCATTACTAGAACGTGTATACGGTGCCATCTTCGAAATAGA**A/C**CCAGAACCTTCGATGTATGG	—	—

Sequence reads are the 64-bp reads about the SNP marker, as returned from the UNEAK pipeline; in bold: <reference allele>/<alternate allele>. Homologous sequences are significant matches to the *Brachypodium distachyon* genome sequence (v1.0), found by BLAST (Altschul *et al.* 1997) in the Gramene database (http://www.gramene.org; Jaiswal *et al.* 2006).

## Discussion

### Genotype calling uncertainty

Genotype calling uncertainty in GBS typically arises from sequencing error, which generates miscalls in SNP alleles, and low sequencing depth, which causes heterozygotes to be miscalled as homozygotes. Genotype calling uncertainty may be accounted for by applying cut-offs on proportion of reads (*e.g.*, calling individuals homozygous for a SNP if >80% of their reads is of a particular allele) or probabilistic methods (returning posterior probabilities of genotypes), which rely on some estimates of error rate and population allele frequency at SNPs (Nielsen *et al.* 2011). In this study, sequencing depth was very low; there were, on average, 0.29 reads per SNP called (SD across SNPs: 0.38). As a result, none of the aforementioned methods could be used conveniently (probabilistic methods were not used because of the high amounts of missing values, particularly prohibitive for estimating population allele frequencies). Genotype calling uncertainty was therefore not accounted for, and the genotypes were used as returned by the UNEAK pipeline in the imputation procedure and association tests. Because genotype calling uncertainty translates into random error under a GWAS model, not accounting for it typically results in shrunk marker effects and loss of power (Nielsen *et al.* 2011). Because there was presumably no systematic loss in precision (true discovery rate), any GWAS inference made from (CC) or (MI*) was still deemed valid in this study. Nonetheless, false positives may have arisen at random from noise (due to genotype-calling uncertainty or other sources), particularly in (CC), which usually had low sample size $n_{obs}$.

### The use of tree models for imputation

Here, no reference genome or genetic map was available for imputing the GBS marker data. Although it was possible to generate genetic maps on LP1 and LP2, the proportion of missing values was such that, in both populations, parental SNP genotypes could not be determined clearly (in particular, 37.6% and 25.6% of selected markers had missing values at both parents in LP1 and LP2, respectively) and imputation uncertainty would have to be accounted to produce reliable genetic maps, which would have been a serious statistical and computational challenge. Consequently, imputation methods based on HMM, which can be very accurate, were not used. In presence of unordered marker data with a general pattern of missingness and no reference panel, the strategy described here to impute missing values (*i.e.*, FCS based on tree models), should be pertinent: although computationally intensive, the proposed imputation procedure was flexible, easy to implement, and appropriate for modeling marker data, as was suggested by Dai *et al.* (2006).

### Structure in the panels

Analysis of structure in the three panels combined revealed stratification by panels, race, and geographical origin. The observed stratification by race and geographical origin was consistent with the results from Jakubowski *et al.* (2011), *i.e.*, *P. caesia* being genetically distinct from *P. arundinacea* and, within *P. arundinacea*, East European strains being distinct from West European and North American strains. Even though accessions native to North America with a genetic background distinct from that of European accessions do exist (Jakubowski *et al.* 2012), most accessions of reed canarygrass found in North America, including all those evaluated in this study, share some common ancestry with West European accessions.

### Results of imputation-based association tests

In this association study, for the rare cases in which *γ* was low enough (γ<0.30), there were gains of significance from (CC) to (MI*) (and, presumably, a gain of power) (Figure 7A, Table 7). Another interesting outcome from MI was the decreased significance, given one marker (TP477925), for only a subset of the associations detected in (CC) (Table 7). Such results suggest a possible gain of precision in MI-based association tests. Unfortunately, no novel significant markers could be detected from (CC) to (MI*). As expected, for high values of *γ* (γ>0.30), MI had the desired property of decreasing significance of associations for the sake of precision.

Values of *γ* lower on average than 0.3 would correspond to PMV lower than approximately 0.45 according to the model presented in Table 6, with values of MAF and AMI set to 0.1 and 0.05, respectively. PMV lower than 0.45 corresponded to 1.6%, 1.8%, and 1.6% of the markers considered for GWAS in the LP1, LP2, and AP-4x subsets, respectively. As discussed below, if a reference panel had been available for imputation, then there would have been many more opportunities for gains in power from MI.

### GWAS results and similarity of marker sequences to *Brachypodium distachyon*

This GWAS revealed associations of markers with multiple traits, which may indicate pleiotropy of one single tagged causal variant, LD between distinct causal variants affecting different traits, or genetic correlation among traits. For example, TP87762 was negatively associated with TSC and positively associated with Ds, St, ARA, and Mg. This result would probably suggest that, through lower tiller density, reed canarygrass plants carrying the nonreference allele at TP87762 were less prone to disease and lodging and had higher concentration of arabinose and magnesium.

Significant markers could not be mapped to a particular region of the reed canarygrass genome, because no reference map or genome sequence is available for that species. However, the significant markers identified here may be used in further studies making use of DNA sequences in reed canarygrass. Potentially, our studies may bring some insight about the function of genes in related species such as Brachypodium or oat. However, actual causal genes were probably not directly tagged by significant markers. Markers that did not map to the Brachypodium genomes (TP140584, TP191264, TP217634, TP477925, TP521945, and TP87762; because of absence of homology or because of evolutionary divergence) were probably in LD with unmarked functional regions, which possibly could have been mapped to the Brachypodium genome. TP184396 and TP 341988 could be mapped to the Brachypodium genome, but within genes with very general purposes (both matched genes involved in mRNA translation). Such results could be “true hits,” but this does not seem very likely. However, TP268059, negatively associated to CP and positively associated to XYL_Eff, was mapped to the exon of a gene coding for Phenylalanine/Histidine-Ammonia-Lyase. Although it is entirely possible that TP268059 did not directly tag the true causal gene, it is plausible that TP268059 is a true hit: Phenylalanine-Ammonia-Lyase (PAL) is involved in the very first step of the monolignol biosynthetic pathway leading to the synthesis of lignin (Boerjan *et al.* 2003), and a decreased ability to synthesize lignin is consistent with lower crude protein content and higher xylose conversion efficiency because lignin inhibits fermentation (Vogel and Jung 2001). However, one would then expect to find significant associations involving Lignin (lignin content) and GLC_Eff (glucose conversion efficiency), which was not the case here. That said, the association between TP268059 and lignification still makes good sense from the homology of the marker in the Brachypodium genome and the relative directions of the detected effects.

### A genome-wide association study based on multivariate MI

Historically, MI has been prevalent in epidemiological studies, probably because of the frequent and prohibitive occurrence of nonresponse in survey data (Rubin 1987; Rubin 1996; Klebanoff and Cole 2008; Sterne *et al.* 2009). Here, the large amount of missing values in our GBS data motivated us to use MI to account for imputation uncertainty and make sound inferences about marker-trait associations. There have been previous quantitative genetics studies that used MI to increase the precision of significance tests for marker-associated effects: Dai *et al.* (2006) used an imputation procedure similar to that presented here and compared it with expectation-maximization techniques. However, their study dealt with small marker data (10 SNPs in real data and 4 SNPs in simulated data) and limited PMV (10% or 20% of missing data). Bobb *et al.* (2011) used MI to gain precision in QTL mapping, but they generated multiple imputes of the phenotypic data, not the genomic data. To our knowledge, this study is the first report of MI applied in a genome-wide context (with thousands of markers over the genome). We believe the methodology presented here could be useful in large-scale genetic analyses involving hypothesis testing in two ways. First, it exemplifies the use of tree models to impute unordered marker data in GWAS and, in the context of multivariate MI, approximate the distribution $p\left({\hat{X}}_{\mathrm{mis}}|X_{\mathrm{obs}}\right)$ by following the methodologies developed by Dai *et al.* (2006) and Burgette and Reiter (2010). As multiplexed GBS, which trades sequencing depth (and therefore genotyping costs) for uncertainty in genotype calling and imputation, is increasingly used for association mapping (Poland and Rife 2012), this type of imputation procedures in a MI context should be particularly useful in species where no or only part of a reference genome is available, like wheat or switchgrass. Second, it shows how estimates from different imputes about marker effects in the unified linear mixed model (Yu *et al.* 2006) could be pooled using the rules developed by Rubin (1987) to conveniently account for imputation uncertainty and perform statistically valid association tests. That said, MI is not the only method that has been developed to explicitly account for imputation uncertainty. A basic approach would be to use expected genotype counts based on some distribution $p\left({\hat{X}}_{\mathrm{mis}}|X_{\mathrm{obs}}\right)$ and to perform association tests as if the marker data were fixed, as was done in (AD) here (Guan and Stephens 2008; Zheng *et al.* 2011). As noted previously (Guan and Stephens 2008), such simplification may yield erratic behavior in association testing if imputation uncertainty is high *and* marker effects are large, which is consistent with the results obtained here (Figure 3B). A more sophisticated approach would be to use tests with explicit account for increased variance of parameter estimates due to imputation uncertainty that are based on some approximation of $p\left({\hat{X}}_{\mathrm{mis}}|X_{\mathrm{obs}}\right)$ (Marchini *et al.* 2007; Guan and Stephens 2008; Zheng *et al.* 2011). In this framework, frequentist tests include score tests and likelihood ratio tests, implemented in SNPTEST (Marchini *et al.* 2007). The Bayesian counterparts of this type of tests, implemented in SNPTEST and BIMBAM (Servin and Stephens 2007), feature some interesting advantages compared to the frequentist tests; in particular, they avoid an inflation in significance arising from high imputation uncertainty (Guan and Stephens 2008). Unfortunately, both the frequentist and Bayesian methods described above, in the current state of their implementations, are limited in the type of models that can be fitted to the data: the tests do not apply to linear mixed models (accounting for relatedness through the **K** matrix), and uncertainty in covariates cannot be conveniently accounted for, as was done in (MI*) here. Although in human GWAS such models may be appropriate (*e.g.*, Burton *et al.* 2007), the strong population stratification in plant GWAS panels call for mixed models that incorporate information on population structure and relatedness (Zhu *et al.* 2008). We believe the approach used here was particularly useful in that it allowed accounting for imputation uncertainty when using linear mixed models for testing associations.

### Properness of imputations

An imputation procedure is proper if it is unbiased and it is confidence-valid, *i.e.*, the variability among imputed values is equivalent to what it would be if the data had been complete. Here, we made the MCAR assumption stating that no factor (in particular, not the phenotypes of interest) influenced missingness at the marker data (Rubin and Little 2002; Sterne *et al.* 2009). Because marker effect estimates based on complete cases are unbiased under the MCAR assumption, (CC) analyses served as the reference to assess consistency of imputation-based tests. Judging from regression analyses on marker-effect estimates from (AD) (Table 4) and from (MI) (Table 5), it seemed that imputations did not generate bias, with shrinkage in marker-effect estimates from MI occurring only because of imputation uncertainty in (MI) [and (MI*)], which is desirable. Imputation bias may have occurred if the MCAR assumption—more generally, the MAR assumption—had not been valid. This may occur in survey data but is unlikely to occur, systematically, in GBS data; the probability of a marker read being available is unlikely to vary systematically across individuals. Confidence validity could only be assumed here. For other imputation methods, which do not preserve the variability present in the original dataset, this assumption cannot be made. Such methods, which include mean imputation and major allele imputation, should therefore be avoided in inference studies.

In the future, a simulation study might be conducted to characterize the unbiasedness and confidence validity of our imputation procedure in a setting where both marker effects and missing values are known *a priori*. Similar analyses were already performed on SNP data (*e.g.*, Dai *et al.* 2006), but assessing the properness of MI based on GBS data in a genome-wide context could certainly be useful.

### Practical issues for MI in GWAS

Here, given the amount of missing data (78% on average) and the information available for imputation (no reference panel and no genetic map of markers), imputation uncertainty was too prohibitive for detecting novel significant markers when performing imputation-based association tests. Gains of significance were nonetheless possible, mostly for low values of *γ* (Figure 7A), corresponding approximately to PMV <0.45 (Figure 7B). Pasaniuc *et al.* (2012) showed that a very substantial gain in imputation accuracy on low-depth GBS data in humans could be achieved when using a reference panel (the 1000-genomes data). For example, with 0.1× sequencing depth, they reported an increase in imputation accuracy from approximately 0.05 to more than 0.70 when the reference panel was used. In light of the results from Pasaniuc *et al.* (2012) and this article, we recommend imputation-based association studies on GBS data with sporadically missing values only when coverage is high enough to produce few missing values *or* when a reference panel is available for imputation.

When dealing with marker datasets that are much larger than the one considered here (numbers of markers *q* on the order of $10^{5}$ or $10^{6}$), implementing MI in GWAS would be a challenge. Computational and memory requirements will of course increase and parallelization should be devised accordingly. When dealing with unordered marker data, MI based on CART should be a good choice with regard to computational—and statistical—efficiency. However, the mice package uses a q×q predictor adjacency matrix to keep record of the candidate predictors for each marker. With many threads and/or high *q*, memory usage can be reduced by replacing the adjacency matrix with a $q\times q_{selected}$ adjacency list ($q_{selected}$: number of variables selected as potential predictors; *e.g.*, 500 in this study) or determining the set of candidate predictors at each iteration with no storage, hence trading computational efficiency for memory efficiency. Both of these measures would involve modifications of the mice code. Importantly, if a (completely typed) reference panel **H** is available for imputation, then one can base imputations of $X_{\mathrm{mis}}$ on **H** only, *i.e.*, $p\left({\hat{X}}_{\mathrm{mis}}|X_{\mathrm{obs}},H\right)$ becomes $p\left({\hat{X}}_{\mathrm{mis}}|H\right)$. In such settings, when imputing a given variable *k*, there would be no uncertainty about values at the predictors to account for (supposedly, genotypes or haplotypes in **H** have been perfectly called). As a result, the multiple imputed datasets in MI could be sampled by ordinary MC instead of MCMC. This would dramatically reduce the computational and memory requirements when implementing MI. Note also that when imputing at markers that are completely untyped in **X** (*in silico* genotyping), basing imputations on $p\left({\hat{X}}_{\mathrm{mis}}|H\right)$ rather than $p\left({\hat{X}}_{\mathrm{mis}}|X_{\mathrm{obs}},H\right)$ may actually yield more accurate imputations (Guan and Stephens 2008). Thus, in presence of a reference panel, MI should be not only more useful (imputations being more accurate) but also more tractable (probably applicable when dealing with hundreds of thousands of markers).
